# Comparison of protein interaction networks reveals species conservation and divergence

**DOI:** 10.1186/1471-2105-7-457

**Published:** 2006-10-17

**Authors:** Zhi Liang, Meng Xu, Maikun Teng, Liwen Niu

**Affiliations:** 1 Hefei National Laboratory for Physical Sciences at Microscale and School of Life Sciences, University of Science & Technology of China, 96 Jinzhai Road, Hefei, Anhui 230027, China; 2 Key Laboratory of Structural Biology, Chinese Academy of Sciences, 96 Jinzhai Road, Hefei, Anhui 230027, China

## Abstract

**Background:**

Recent progresses in high-throughput proteomics have provided us with a first chance to characterize protein interaction networks (PINs), but also raised new challenges in interpreting the accumulating data.

**Results:**

Motivated by the need of analyzing and interpreting the fast-growing data in the field of proteomics, we propose a comparative strategy to carry out global analysis of PINs. We compare two PINs by combining interaction topology and sequence similarity to identify conserved network substructures (CoNSs). Using this approach we perform twenty-one pairwise comparisons among the seven recently available PINs of *E.coli*, *H.pylori*, *S.cerevisiae*, *C.elegans*, *D.melanogaster*, *M.musculus *and *H.sapiens*. In spite of the incompleteness of data, PIN comparison discloses species conservation at the network level and the identified CoNSs are also functionally conserved and involve in basic cellular functions. We investigate the yeast CoNSs and find that many of them correspond to known complexes. We also find that different species harbor many conserved interaction regions that are topologically identical and these regions can constitute larger interaction regions that are topologically different but similar in framework. Based on the species-to-species difference in CoNSs, we infer potential species divergence. It seems that different species organize orthologs in similar but not necessarily the same topology to achieve similar or the same function. This attributes much to duplication and divergence of genes and their associated interactions. Finally, as the application of CoNSs, we predict 101 protein-protein interactions (PPIs), annotate 339 new protein functions and deduce 170 pairs of orthologs.

**Conclusion:**

Our result demonstrates that the cross-species comparison strategy we adopt is powerful for the exploration of biological problems from the perspective of networks.

## Background

The activity of cellular life relies on properly functioning of the extremely complex interaction networks among numerous intracellular constituents. The analysis of the topology and dynamics of these networks within a living cell offers a new window to explore the problems relating principles on the construction, function and evolution of life [1]. Progress in identifying the protein-protein interactions (PPIs) within the protein interaction networks (PINs) has furnished us with powerful high-throughput approaches, such as the two-hybrid assay [2], affinity purification [3], protein chips [4] and phage display [5], as well as computational methods [6,7]. To date, these technologies have generated large PINs for several model organisms, such as *H. pylori *[8], *S. cerevisiae *[9,10], *C. elegans *[11] and *D. melanogaster *[12] and large amount of data has been deposited in publicly accessible databases, including DIP [13], BIND [14], MINT [15] etc.

Both opportunities and challenges are present in the study of molecular interaction networks. High error rate in high-throughput data requires the enhancement of our abilities in discrimination of true PPIs from false positives [16] as well as data collection to avoid false negatives. Network topology information can be used to predict protein functions [17] and reformulate old questions from a network perspective [18,19]. Besides, studies on complex networks have uncovered unexpected nonrandom global organizational patterns, some of which also exist in PINs. One of the most significant features is the scale-free organization of PINs [11,12,20,21]. The scale-free topology is associated with the ability of resilience against components failure and environment changes [21,22]. To address the possible mechanisms in the development of scale-free structure of real PINs, several models based on gene duplication and divergence have been proposed [23,24]. It was also found that signatures of hierarchical modularity are present in PINs [12,20], which urges objective definition and automatic identification of topological and functional modules [25-27]. In addition, recent decomposition of PINs into motifs discloses some specific patterns of PINs at the local level [28,29].

**Figure 1:**
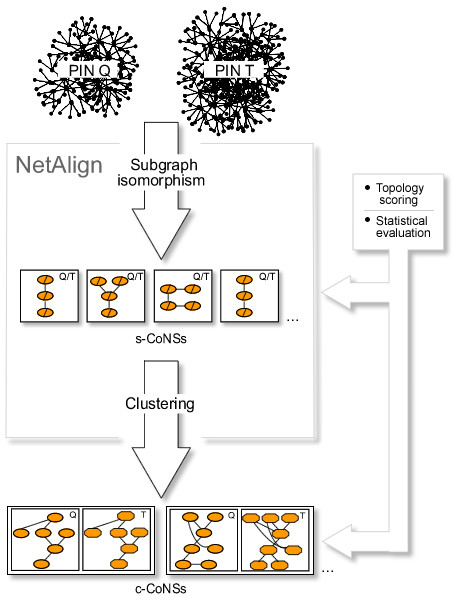
**Schematic of pairwise network comparison in NetAlign**. The comparison between two PINs is accomplished by a fast subgraph isomorphism algorithm and the resulting s-CoNSs are connected maximal common subgraphs (MCS) and exact matches of the two networks. The s-CoNSs are further merged by a clustering rule to produce c-CoNSs that allow inexact match among homologous regions of interaction in the two networks. The identified s-CoNSs and c-CoNSs are scored on the basis of their interaction topologies and evaluated by statistical significance.

**Figure 2:**
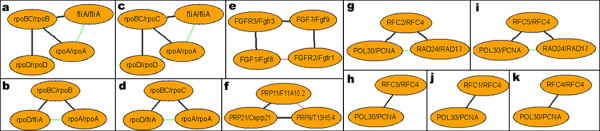
**Representative s-CoNSs**. Each pair of matched conserved proteins from two different species is shown in one node with their identifiers delimited by a slash; black edges are conserved PPIs existed in both PINs and constitute the s-CoNS, while red/green edges are discrepant PPIs observed only in the species on the left/right of the slash, respectively. **a-d. **These s-CoNSs corresponds to the RNA polymerase (RNAP) of prokaryotes and are identified from the PIN comparison between *E.coli *(left) and *H.pylori *(right) (figure 10). **e. **This s-CoNS is from the NetAlign analysis between *H.sapien *and *M.musculus*. It is a part of the system of fibroblast growth factors (FGF) and FGF receptors (figure 8). Gene duplication present in this system results in great redundancy for the identified s-CoNSs that 151 very similar s-CoNSs are identified. **f. **This is an s-CoNS harbored by the PINs of *S.cerevisiae *and *C.elegans*, and it is a part of E2F/DP transcription factor complex (figure 4). Based on the discrepant red edge, we predict that F11A10.2 interacts with T13H5.4 and this prediction is also present in the interolog database [32]. **g-k. **These s-CoNSs constitute the complex of replication factor C (figure 3) and are derived from the comparison between the PINs of *S.cerevisiae *and *H.sapien*.

**Figure 3:**
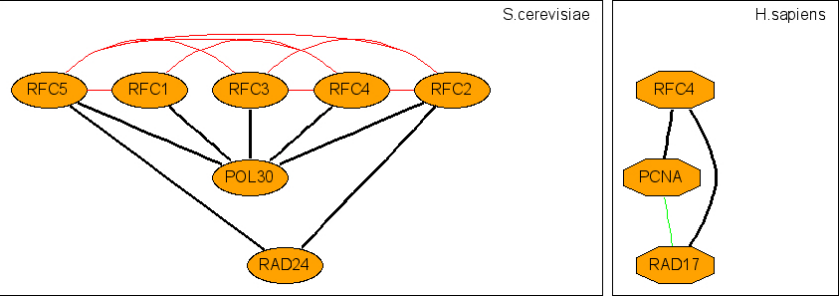
**Representative c-CoNS: the complex of replication factor C (RFC)**. Figure 3-10 are representative c-CoNSs. Each c-CoNS is shown in two separate panels each for a species; orthologous and transitively orthologous proteins are shown in the same horizontal level in each panel. Black edges are conserved PPIs existed in both PINs and constitute c-CoNSs, while red/green edges are discrepant PPIs observed only in the species on the left/right panel, respectively.

**Figure 4:**
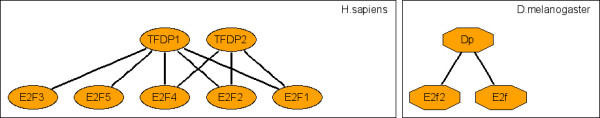
Representative c-CoNS: E2F/DP transcription factor complex.

**Figure 5:**
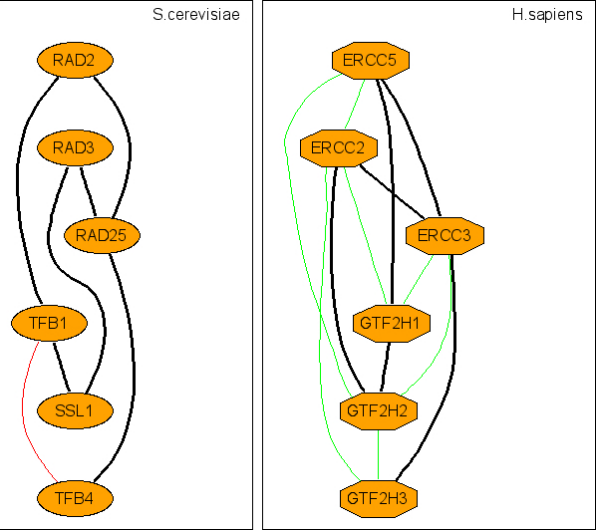
Representative c-CoNS: the general transcription and DNA repair factor IIH (TFIIH) complex.

**Figure 6:**
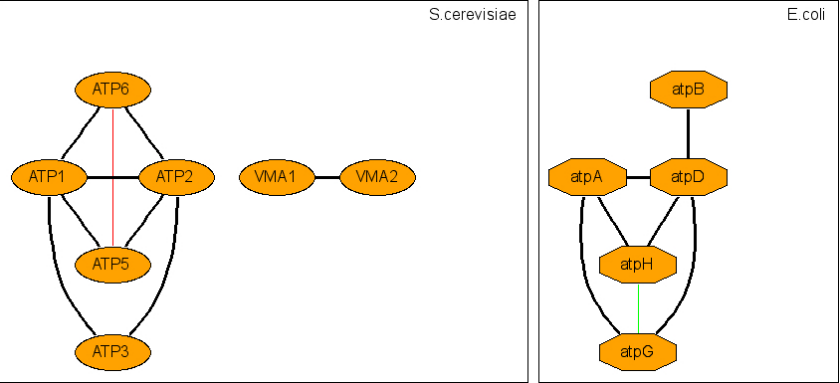
Representative c-CoNS: ATP synthase.

**Figure 7:**
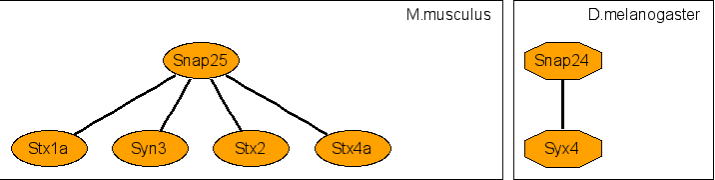
Representative c-CoNS: synaptosomal neurotransmitter release.

**Figure 8:**
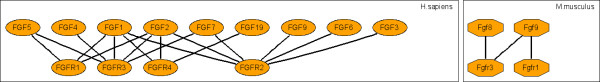
Representative c-CoNS: system of fibroblast growth factors (FGFs) and FGF receptors.

**Figure 9:**
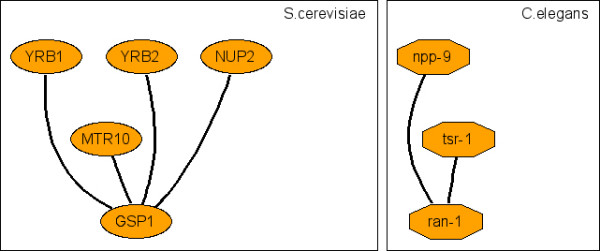
Representative c-CoNS: nucleocytoplasmic transport.

**Figure 10:**
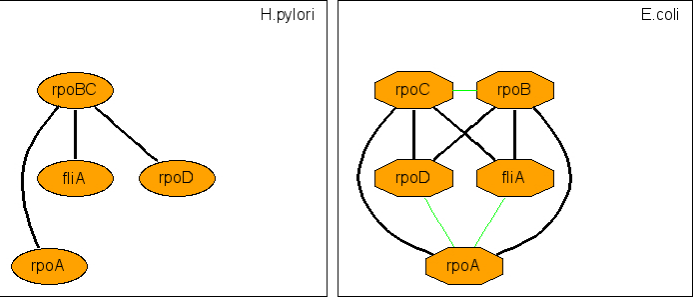
Representative c-CoNS: bacterial RNA polymerase (RNAP).

As a powerful method, cross-species comparison often provides insights into the underlying laws behind complex biological phenomena. Motivated by this we propose an efficiently computational strategy called NetAlign to enable the comparative analysis of two PINs. NetAlign searches for conserved network substructures (CoNSs) that can pair in two PINs by integrating information on interaction topology and protein sequences. It implements a modified graph comparison algorithm and a clustering rule to accomplish pairwise comparison of PINs, and includes two processes for scoring and evaluating the identified CoNSs (figure 1). We apply the NetAlign method to the seven PINs of *E. coli*, *H. pylori*, *S. cerevisiae*, *C. elegans*, *D. melanogaster*, *M. musculus *and *H. sapiens *and perform twenty-one genome-scale pairwise comparisons among them (figure 2, 3, 4, 5, 6, 7, 8, 9, 10). We show that beyond what is gleaned from the genome, PIN comparison not only reveals species conservation but also indicates potential species divergence at the PIN level. And the identified CoNSs are known or candidate conserved complexes and can be used to predict PPIs, protein functions and orthologs.

## Results

### Conservation of PINs

As seen from the twenty-one pariwise comparisons, PINs have only minor overlap (Table 1). This attributes to the incompleteness of data and the difference among species. We introduce an overlap score to evaluate the overlap between any two PINs N_Q _and N_T_. The overlap score is defined as (Q_C_/Q_0_+T_C_/T_0_)/2, where Q_C _is the number of conserved PPIs in N_Q _derived from the comparison between N_Q _and N_T_, Q_0 _is the the number of PPIs in N_Q_; T_C _and T_0 _are their counterparts in N_T_. This score ranges from 0 (i.e. N_Q _and N_T _never overlap) to 1 (i.e. N_Q _and N_T _overlap completely). Obviously, given complete interaction data, the overlap score can quantify species conservation from the view of PIN. Even in case of poor data, some implications can also be obtained. Given that the observed PPIs are from random sampling of real PINs, the overlap score can still reflect the conservation between PINs to some extent. It seems that close species would have larger overlap. For instance, although the two bacterial PINs are not so large, they overlap with each other more than with some other larger PINs such as that of *D.melanogaster*; another example is the significant overlap between the PINs of mouse and human, both of which are nearly the smallest among the seven. In addition, there is an obvious decrease in the number of identified c-CoNSs compared with that of identified s-CoNSs and it suggests great redundancy exists in s-CoNSs. In fact, this results from gene duplication and divergence that make many small and local duplicated interaction topologies in PINs.

**Table 1:** Overview of the twenty-one pairwise comparisons of PINs.

	E.coli	H.pylori	S.cerevisiae	C.elegans	D.melanogaster	M.musculu	H.sapien
*E.coli*	-	0.020	0.026	0	0.009	0	0
*H.pylori*	7/3	-	0	~0	0	0	0
*S.cerevisiae*	19/8	1/1	-	0.010	0.020	0.082	0.064
*C.elegans*	0/0	1/1	103/32	-	0.005	0	0
*D.melanogaster*	8/3	0/0	358/101	114/70	-	0.044	0.073
*M.musculu*	0/0	0/0	164/7	5/3	24/13	-	0.309
*H.sapien*	0/0	0/0	109/23	7/6	112/18	504/25	-

The upper triangle displays overlap scores of pairwise PIN comparisons. The lower triangle shows the number of identified s-CoNSs over the number of identified c-CoNSs of each such comparison.

**Figure 11:**
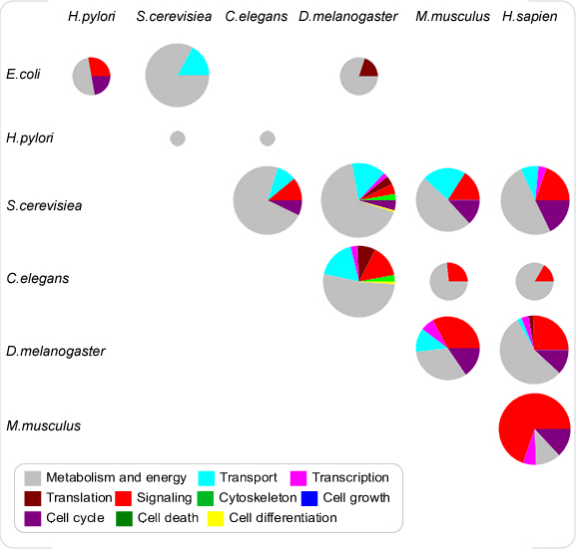
**Function distribution of the identified CoNSs**. Each pie chart represents the distribution of the ten functional categories of the CoNSs derived from a pairwise PIN comparison. The area of each pie chart is approximately scaled according to the number of conserved proteins involved in the CoNSs.

What are the identified CoNSs with regard to? One way to answer this question is to inspect their functions. We associate proteins with their known biological functions using the Gene Ontology annotations (GO; Oct 2005 version; [31]) and analyze the GO annotations within CoNSs. Due to the hierarchical structure of GOs, for each protein we propagate its GO annotations upwards through the GO hierarchy and retrieve all the relevant GO annotations. We define that a CoNS to be functionally homogenous, if it contains at least a GO annotation that satisfies the following conditions: (1) for either of the corresponding two species, at least half of its proteins in the CoNS have this GO annotation; (2) the annotation is sufficiently specific, namely at least at GO level four from the root of the GO hierarchy. It is found that more than 80 percent of the CoNSs are homogenous, that is, CoNSs are also functionally conserved across species. Furthermore, to get an estimation of the function distribution of the CoNSs derived from a pairwise PIN comparison, we consider ten functional categories concerning cellular function selected from top levels of the GO hierarchy. For each CoNS, the most frequent function categories satisfying the above conditions are assigned to every protein in it. Then the function categories assigned in all the CoNSs are pooled together and the frequency of each function category is computed. We find that the most plenty functions are related to cellular metabolism and energy, and the functions involving in transport, signaling and cell cycle are also abundant (figure 11).

### Divergence of PINs

Species divergence is usually studied in terms of genomes. However, it is obvious that species divergence must also be present at the level of PINs. Here, by virtue of CoNS difference between species, we probe the conservation of the interaction topology of orthologs across species. Since s-CoNSs are exactly matched subnetworks, it indicates that different species harbor many locally conserved interaction regions that are topologically identical. Many s-CoNSs are almost the same except for minor differences due to matching permutations and it reflects the duplication of genes and interactions. On the other hand, many of the matched c-CoNSs of different species show that although they have similar framework of interaction topology, their detailed topological organizations can be different. This also arises from duplication and divergence of genes and the associated interactions. For instance, the RNA polymerase (RNAP) identified from the PIN comparison between *E.coli *and *H.pylori *(figure 2a–d, 10) shows difference of the two bacteria in transcription. Four very similar s-CoNSs with minor matching differences constitute the corresponding c-CoNS of the RNAP. It suggests that the symmetric interaction topology of the *E.coli *RNAP results from a duplication event and the RNAP of *H.pylori *lacks this duplication and serves as a prototype of this molecular machine. So it seems that homologous local regions of interaction which are topologically identical are popular across species and these regions constitute larger interaction regions that are topologically different but similar in different species. In addition to our above analysis of function homogeneity, it is conjectured that different species achieve similar or the same biological functions by organizing orthologs in a similar but not necessarily the same interaction topology. Theoretically, any species-to-species difference in c-CoNSs discloses the difference of the corresponding two species in some aspect. Currently, however, due to the incompleteness of data, some of the identified differences may be false. But with the fast growth of data, our method offers a way to discover species difference and explore the problem of species divergence at the network level.

### CoNSs vs. complexes

During the analysis of the identified CoNSs, another question concerns us: to what extent do the CoNSs overlap conserved complexes or pathways? In order to give a rough estimate of this, we use the MIPS yeast complex repertoire as a reference to evaluate the identified yeast c-CoNSs derived from the six pairwise PIN comparisons between yeast and the other species. Only those MIPS complexes that are manually annotated independently from the DIP data are considered, that is, we exclude all the complexes in MIPS category 550 that are based on high-throughput experiments. We compare the c-CoNSs with the reference complexes, and if the proportion of the intersecting proteins between a yeast c-CoNS and a MIPS complex exceeds a threshold the c-CoNS is accepted as a hit. Under the 80% overlap threshold, 70 hits concerning 61 c-CoNSs are found, which accounts for about 35% of the 172 yeast c-CoNSs (Table 2).

**Table 2:** Representative result of comparisons between yeast c-CoNSs and MIPS complexes.

S.cerevisiae *vs*.	*No. of hits*	*No. of involved c-CoNSs*	*Representative results*				
			
			c-CoNS	Overlap proportion	MIPS complex	Overlap proportion	No. of common proteins
*E.coli*			5	100%	70	100%	2
	6	5	6	100%	430	50%	2
			8	100%	80	100%	2
*H.pylori*	0	0	0	0	0	0	0
*C.elegans*			1	100%	410.40.30	100%	5
			6	100%	110	100%	4
	11	10	8	67%	440.30.10.20	100%	2
			18	100%	177	40%	2
*D.melanogaster*			2	83%	140.10.20	71%	5
			3	100%	360.10.10	47%	7
			5	100%	410.40.30	100%	5
			13	100%	120.2	100%	4
	40	34	26	100%	500.10.30	60%	3
			58	100%	500.10.30	40%	2
*M.musculus*			2	31%	470.20	80%	4
	3	3	3	100%	133.10	60%	6
			5	100%	510.160	75%	3
*H.sapien*			1	83%	510.180.10.30	56%	5
			2	100%	133.10	60%	6
	10	9	3	71%	410.40.30	100%	5
			13	100%	510.70.20	25%	3

It is found that some c-CoNSs correspond to the whole complexes, some are parts of a certain complex and some overlap several different complexes. For instance, c-CoNS 1 from *S.cerevisiae *vs. *C.elegans *completely overlaps MIPS complex 410.40.30, the DNA replication factor C that consists of five subunits RFC1, RFC2, RFC3, RFC4 and RFC5 (this complex is also identified from the comparisons of *S.cerevisiae *with *D.melanogaster *and *H.sapien*); c-CoNS 26 and c-CoNS 58 from *S.cerevisiae *vs. *D.melanogaster *compose the entire MIPS complex 500.10.30, the translation initiation factor (eIF), and the former contains three subunits GCD7, GCN3 and GCD2, the latter includes the remaining two subunits GCD6 and GCD1; part of c-CoNS 2 from *S.cerevisiae *vs. *M.musculus *overlaps four proteins STE7, KSS1, STE11 and FUS3 out of the five proteins of MIPS complex 470.20, a complex involved in the activation of MAP kinase (MAPK) in the Ras pathway. These demonstrate the validity of cross-species comparison for identifying conserved functional modules in PINs and the non-hit c-CoNSs may be candidate complexes or pathways for experimental validation.

### Prediction of PPIs

Based on the cross-species conservation of CoNSs, there are two ways to make use of the conserved PPIs in the identified CoNSs (Table 3). The first is rather simple. A conserved PPI observed in two species is probably also present in the third species, especially when the three species belong to the same evolutionary branch. Such-and-such, a conserved PPI observed in more species is more likely to appear in other species. Totally, we collect 1178 conserved PPIs (additional file 1). These PPIs are useful references to check newly observed PPIs and can be transferred to other species. The second is also intuitive. Due to the conservation of CoNSs, discrepant PPIs (see red or green edges in figure 2, 3, 4, 5, 6, 7, 8, 9, 10 for examples) that are formed by conserved proteins in a CoNS but exist in only one of the two species have a high probability to be also present in the other species. Operationally, we use s-CoNSs to make predictions. Given an s-CoNS derived from the comparison between two PINs N_Q _and N_T_, as well as conserved proteins A_Q_, B_Q _of N_Q _and their counterparts A_T_, B_T _of N_T _in the s-CoNS, if A_T _and B_T _do not interact, but A_Q _and B_Q _interact, then the interaction A_Q_-B_Q _is transferred to A_T_-B_T _(see figure 2f for an example). At last, 101 new PPIs are predicted (additional file 2).

**Table 3:** Conserved and predicted PPIs.

	E.coli	H.pylori	S.cerevisiae	C.elegans	D.melanogaster	M.musculus	H.sapien
The number of conserved PPIs	33	8	367	122	285	128	235
The number of predicted PPIs	2	2	12	1	14	36	34

The first row shows the number of conserved PPIs of each species derived from the identified CoNSs. The second row shows the number of new PPIs predicted on the basis of discrepant PPIs formed by conserved proteins in CoNSs.

On the whole, our method is similar to the prediction of PPIs from interologs that are defined to be orthologous pairs of interacting proteins in different organisms [32]. However, the two methods are different in determining whether a PPI can be transferred. The latter method transfers a PPI between species on the basis of the joint sequence similarity of the corresponding two pairs of interacting proteins, while our method transfers a PPI based on the conservation of local interaction topology between species. The current interolog database includes predicted PPIs for *C.elegans *and *D.melanogaster*. We compare our predictions with them and find that our only one prediction for *C.elegans *is collected in the database but the fourteen predictions for *D.melanogaster *are not present. It is natural that the two methods can intersect, since the conservation of sequences and the conservation of interactions are consistent sometimes. However, a PPI discarded by the interolog method may also be supported by our method if it is part of a high score CoNS. So, to some extent, our method is complement of the interolog method.

### Prediction of protein functions

We have seen that CoNSs are functionally homogenous and have significant coverage with known complexes. So it is natural to guess that if many proteins in a CoNS have the same function, the remaining proteins would also have that function. Based on this idea, we strictly analyze the GO annotation enrichment in c-CoNSs with a *p*-value < 0.001 and predict new protein-GO annotation associations whenever the following conditions are satisfied: (1) the set of proteins in a c-CoNS is significantly enriched for a particular GO annotation (*p*-value < 0.01); (2) the GO annotation satisfies the conditions for functional homogeneity. Then for both species, all remaining proteins in the c-CoNS are predicted to have the enriched GO annotation.

To assess the overrepresentation of a GO term, we compute a *p*-value of significance by a hypergeometric test that answers the question: when sampling X proteins (the set of c-CoNS proteins) out of Y proteins (the set of proteins of the species), what is the probability that x or more of the X proteins belong to a GO functional category shared by y of the Y proteins? To control the rate of false positive, the *p*-value is further Bonferroni corrected for multiple testing. The analysis of eukaryotic c-CoNSs gives 339 predictions of protein-GO annotation associations (additional file 3).

### Discovery of orthologs

Orthologs are proteins in different species that evolved from a common ancestor by speciation and they are often deemed as having the same or similar biological functions. An important aspect of protein functions is the physical interactions of proteins with other molecules, in particular, with other proteins. Based on the concept that similarity in interaction topology may indicate similarity in function and thus orthologs, we deduce orthologs. In our prediction, we only consider s-CoNSs with a *p*-value < 0.001 and containing at least three conserved PPIs as acceptable orthologous local interaction regions, and take paired proteins as potential orthologs. Finally, we predict 170 pairs of orthologs that are not reciprocally best BLAST hits (additional file 4). We then compare our predictions with the Inparanoid database that collects pairwise ortholog groups of eukaryotes [33], and find that 23 of our 159 predictions on eukaryotes are present in it. To some degree, this result reflects the validity of our method. Clearly, by combining the conservation of interaction topology and sequences our method can make up for some true orthologs ignored by traditional methods.

## Discussion

A related method that performs pairwise network alignment between species is the PathBLAST method [34-36], which offers a general solution to the problem of PIN comparison. This method searches for small seed linear high-scoring alignments and aggregates them by dynamic programming. The decomposition of problem by PathBLAST into sub-problems is expensive in time, although each sub-problem can be solved in linear time. This fact limits its online application so that the PathBLAST server restricts a query to small scale (with no more than 5 proteins and 4 PPIs) linear topology and focuses on the identification of conserved protein interaction paths. Here, we take a completely different way. The core of our NetAlign method is subgraph isomorphism, in our case that is the identification of connected maximal common subgraphs (MCSs) of two PINs, and the followed clustering. In principle, subgraph isomorphism is NP-hard and cannot be solved for arbitrarily large networks. However the actual constraints on PIN comparison, such as limited sizes of PINs and ortholog correspondence, confine the solution space of the problem. In addition, the time-consuming and repetitious operations in searching for disconnected MCSs are avoided, which reduces the recursion tree during the search greatly. All of these make the solution of genome-scale PIN comparison feasible and efficient. The server supported by the NetAlign strategy can accept an arbitrarily connected query PIN and searches a target PIN for CoNSs with arbitrarily topological organization [37]. These features widen its application. The resulting s-CoNSs and c-CoNSs tell us different information on PINs as shown at above. The PathBLAST method allows gaps and mismatches in the alignments, while ours don't. Considering the relative poor quality of current data, we concern ourselves with more conserved local interaction topology and aim to identify conserved interaction regions that are highly confident. Our method circumvents related fuzzy matching problem by clustering and the discrepant PPIs reported are actually gaps, but they do not participate in the solving procedure as in PathBLAST. On the whole, NetAlign and PathBLAST are different solutions to the same problem. By virtue of their different design philosophy and principle, they have different advantages.

It is well known that high-throughput data suffer errors, such as false positives and false negatives. However, our comparative strategy is not sensitive to this kind of noise. As described in the methods section, the identified CoNSs are filtered according to the statistical significance of their scores. This process prefers CoNSs with a non-random-like configuration and size, and effectively decreases the impact of random errors. Here, we give a simple estimation of the impact of false positives. Suppose the *p*-value cutoff of the statistical filter is p, the fractions of false positives of the two compared PINs are q_+ _and t_+_, respectively. For the two cases that lead to errors, namely two false positives match each other and a false positive matches a true positive, their probabilities are q_+_t_+ _and q_+_(1-t_+_)+(1-q_+_)t_+_, respectively. Taken together, p(q_+_+t_+_-q_+_t_+_)^n ^gives the probability that a CoNS with n false conserved edges occurs in the result. In our analysis, only those CoNSs with a *p*-value < 0.05 are taken into account, that is p = 0.05; according to a recent estimation [16], q_+_≈0.5, t_+_≈0.5; so, the probability that a wrong conserved edge occurs is less than 4 percent. Considering the rapid damp of the probability of error occurrence with n, it is obvious that our method is reliable even under high fraction of false positives. As for false negatives, since discrepant PPIs in CoNSs are shown as color edges, it facilitates the identification of them and thus reduces their impact. As a vivid demonstration, we perform six additional pairwise comparisons between a larger *S.cerevisiae *PIN derived from the DIP 20050126 release and the above PINs of the other six species. The result is almost the same as that of the yeast core subset, except that 34 new PPIs of yeast and 27 new PPIs of other species are involved (data not shown). Comparing with its size that is of 4770 proteins and 15199 PPIs and about double size of the core yeast PIN, the difference is negligible. It is obvious that cross-species PIN comparison provides a robust way to analyze PPIs.

Furthermore, what we talk about here is only two-way comparison, an extension to n-way (n > 2) comparison is needed to identify CoNSs across multiple species. For instance, the E2F/DP transcription factor complex is identified in all the three pairwise comparisons among *H.sapien*, *M.musculus *and *D.melanogaster *(figure 4) and the complex of replication factor C (RFC) is also discovered in the pairwise comparisons among *S.cerevisiae*, *C.elegans*, *D.melanogaster *and *H.sapien *(figure 3). These essential molecular machines are highly conserved across species. The n-way extension of the current method will shed light on these conserved interaction topologies and give more reliability as well as conservation on PPI evaluation.

## Conclusion

We propose a computational strategy to perform genome-scale comparative analysis of PINs and apply this approach to the seven largest PINs currently available. In spite of the incompleteness of data, PIN comparison enables us to identify species conservation and divergence present at the network level. We find that the identified CoNSs are conserved not only in topology, but also in function. And the detailed investigation of the yeast CoNSs shows that many of the CoNSs correspond to complexes. Besides, based on the species-to-species difference in CoNSs, we infer potential species divergence. We find that different species harbor many conserved interaction regions that are topologically identical and these regions can constitute larger interaction regions that are topologically different but similar in framework. So it seems that different species organize orthologs in similar but not necessarily the same topology to achieve similar or the same function. To exemplify the application of the identified CoNSs, we reformulate the problems of PPI prediction, function annotation and ortholog assignment from a network perspective. Our result demonstrates that the cross-species comparison strategy we adopt is powerful for the exploration of biological problems in PINs.

## Methods

We develop an efficient computational procedure called NetAlign for comparison of two PINs. NetAlign searches for CoNSs that can pair in two PINs by integrating information on interaction topology and protein sequences. It implements a modified graph comparison algorithm and a clustering rule to accomplish pairwise comparison of PINs, and includes two processes for scoring and evaluating the identified CoNSs (figure 1). We apply the NetAlign method to the seven PINs of *E. coli*, *H. pylori*, *S. cerevisiae*, *C. elegans*, *D. melanogaster*, *M. musculus *and *H. sapiens *and perform twenty-one genome-scale pairwise comparisons among them.

### Preprocessing of PINs

We download data of the seven largest PINs currently available from the DIP. The PIN of *S.cerevisiae *is from the DIP 20041003 core subset that contains validated PPIs in the budding yeast, and the other six are from the DIP 20050126 release. After removing PPIs among different species and self interactions, we obtain the resulting PINs of *E.coli *(398 proteins and 473 PPIs), *H.pylori *(702 proteins and 1359 PPIs), *S.cerevisiae *(2593 proteins and 6272 PPIs), *C.elegans *(2621 proteins and 3951 PPIs), *D.melanogaster *(7025 proteins and 20726 PPIs), *M.musculus *(304 proteins and 250 PPIs) and *H.sapiens *(731 proteins and 805 PPIs).

### Graph model of PINs

In NetAlign, we model a PIN as a labeled, undirected graph N(P,I), where P is a series of vertices representing proteins and I is a set of edges representing PPIs. To compare two PINs N_Q_(P_Q_,I_Q_) and N_T_(P_T_,I_T_) from different species, it is necessary to identify the correspondences of vertices and edges in them. The correspondence between a vertex A_Q _in N_Q _and a vertex A_T _in N_T _is established, in other words, they are labeled the same, if they are putative orthologs. The ortholog relation is determined by a bi-directional BLAST search between the two species, which consists of two BALST searches, one from each direction, both with an E-value ≤ 10^-7^. This removes discrepancy in ortholog assignment arising from a uni-directional BLAST search. The correspondence between a pair of conserved PPIs A_Q_-B_Q _in N_Q _and A_T_-B_T _in N_T _is defined, if A_Q _corresponds to A_T _and B_Q _corresponds to B_T _simultaneously.

### Network comparison

The aim of NetAlign is to identify CoNSs, which may derive from a common ancestor, in two PINs. The identification of CoNSs is naturally formulated as subgraph isomorphism which is a well-know NP-hard problem. To be exact, we take network comparison as enumerating all the maximal common subgraphs (MCSs) in two networks. To avoid meaninglessly repetitious combinations of components in disconnected MCSs during the solution of the problem, we only take connected MCSs into account and define them as s-CoNSs (single CoNSs; see figure 2 for examples). This greatly reduces the searching space of the problem.

To solve the MCS problem of two networks N_Q_(P_Q_,I_Q_) and N_T_(P_T_,I_T_), an edge compatibility graph G = (V,E) is built. Here, V is a set of corresponding edge pairs and is defined as V = {(i_Qm_, i_Tn_) | i_Qm _∈ I_Q_, I_Tn _∈ I_T_, if i_Qm _corresponds to i_Tn_}; E establishes the connection between two edge pairs v_h _= (i_Qa_, i_Ta_) and v_k _= (i_Qb_, i_Tb_), where i_Qa_, i_Qb _∈ I_Q_, i_Ta_, i_Tb _∈ I_T_, as follows: E = {(v_h_,v_k_)| v_h_, v_k _∈ V; if i_Qa _i_Qb _and i_Ta _i_Tb_, and if either i_Qa_, i_Qb _in N_Q _are connected via a vertex corresponding to the vertex shared by i_Ta_, i_Tb _in N_T_, or i_Qa_, i_Qb _and i_Ta_, i_Tb _are not adjacent in N_Q _and N_T_, respectively}. Each complete maximal subgraph in the graph is a MCS between N_Q _and N_T_. The problem is then transformed into an all maximal cliques problem, which requires enumerating all the complete maximal subgraphs. Bron-Kerbosch algorithm is a fast and widely used algorithm for this [30]. Here we implement a variant of this algorithm, which detects all cliques representing connected MCSs.

### Clustering CoNSs

Each identified s-CoNS is a solution of the network comparison and is an exact match between two subnetworks in the two PINs. However, redundancy exists in regions of interaction where paralogs interact and s-CoNSs can overlap each other. Besides, there may be inexact match between the conserved interaction regions in the two PINs due to loss, duplication and divergence of genes and their associated interactions or data incompleteness; and, these regions can be disconnected. In order to handle these, we introduce c-CoNSs (clustered CoNSs; see figure 3, 4, 5, 6, 7, 8, 9, 10 for examples) by merging similar s-CoNSs. Two s-CoNSs are clustered if their number of intersecting vertices is equal to or greater than 80% of the smaller one for either of the two species. Three or more s-CoNSs are clustered by the rule of single linkage, that is, the clustering relation is transitive. If an s-CoNS can not be clustered with others, it forms a c-CoNS itself.

### Scoring strategy

A CoNS is scored based on its size, i.e. the number of conserved PPIs it has, and its connectivity. Each connected component of a CoNS is considered independently and scored as n(n+1)/2, where n is the number of conserved PPIs in it. The ultimate score of the CoNS is the sum of these individual scores. This simple strategy gives higher scores to CoNSs with larger size and better connectivity, since they are more likely to occur not by chance but by conservation in evolution.

### Statistical evaluation

In order to evaluate the statistical significance of an identified CoNS, we compute a *p*-value that is based on the distribution of top scores obtained by applying the above method to randomized data. A PIN is randomized by randomly shuffling the labels associated with the vertices and rewiring the edges but preserving the number of edges of the vertices. We perform 1000 rounds of comparisons between the randomized versions of the two PINs and estimate the *p*-value of a CoNS as the fraction of runs which result in a CoNS with the same or greater score. All the CoNSs taken into account in the analysis followed have a *p*-value < 0.05 unless specified explicitly.

## Availability and requirements

**Project name: **NetAlign

**Project home page: **

**Operating system(s): **Platform independent

**Programming language: **C/C++ and Perl

## Authors' contributions

ZL implemented the NetAlign program and wrote the manuscript. MX wrote programs for data processing. Both of them performed the data analysis. We deem ZL and MX contribute equally to the work. MT and LN supervised the project and helped edit the manuscript. All authors read and approved the final manuscript.

## Supplementary Material

Additional file 1 **Conserved PPIs**. The list of identified conserved PPIs derived from the analysis. Click here for file

Additional file 2 **Predicted PPIs**. The list of predicted PPIs derived from the analysis. Click here for file

Additional file 3 **Function prediction**. The list of predicted function annotations derived from the analysis. Click here for file

Additional file 4 **Ortholog prediction**. The list of predicted orthologs derived from the analysis. Click here for file
