# Supplementary material for: Comparison of protein interaction networks reveals species conservation and divergence
Source: BMC Bioinformatics. 2006 Oct 17;7:457. doi: 10.1186/1471-2105-7-457 (PMC1630707; doi:10.1186/1471-2105-7-457)
Supplement: Additional file 1 — Conserved PPIs. The list of identified conserved PPIs derived from the analysis. [file 1471-2105-7-457-S1.pdf]

	Conserved PPI	*S.cerevisiae*	*M.musculus*	*H.sapiens*	*H.pylori*	*E.coli*	*D.melanogaster*	*C.elegans*
S.cerevisiae	P36008|P32471	v	-	-	-	-	o	-
	P38920|Q12083	v	-	o	-	-	-	o
	P36019|P05986	v	-	-	-	-	-	o
	P32502|P14741	v	-	-	-	-	o	-
	P32457|P32468	v	-	-	-	-	o	-
	P23639|P40303	v	-	-	-	-	o	-
	Q12018|Q08273	v	-	o	-	-	-	-
	Q12199|P23595	v	-	-	-	-	o	-
	P08018|P23561	v	o	o	-	-	-	-
	Q00772|P32490	v	o	-	-	-	-	o
	P32458|Q07657	v	-	-	-	-	o	-
	P08518|P04050	v	-	-	-	o	-	-
	Q07442|P35817	v	-	-	-	-	o	-
	P32628|P33298	v	-	-	-	-	o	-
	P34730|P29311	v	-	o	-	-	-	-
	P21242|P40302	v	-	-	-	-	o	-
	P53941|P47083	v	-	-	-	-	o	-
	P32458|P25342	v	-	-	-	-	o	-
	Q04673|P07276	v	-	o	-	-	-	-
	P52286|Q08273	v	-	o	-	-	-	-
	P15108|P23561	v	-	o	-	-	-	-
	P32598|P40036	v	-	-	-	-	o	-
	Q07350|P32524	v	-	-	-	-	-	o
	Q03337|P38334	v	-	-	-	-	o	-
	P07251|P00830	v	-	-	-	o	-	-
	P04821|P10592	v	-	-	-	-	o	-
	P33313|P02829	v	-	-	-	-	o	-
	P32491|Q00772	v	o	-	-	-	-	o
	P29311|Q03497	v	-	o	-	-	-	-
	Q00245|P38339	v	-	o	-	-	-	-
	P00546|P24871	v	o	o	-	-	o	-
	P07251|Q96101	v	-	-	-	o	-	-
	P22517|P06787	v	-	-	-	-	o	-
	P36047|P33329	v	-	o	-	-	-	-
	P40302|P21243	v	-	-	-	-	o	-
	P21242|P41811	v	-	-	-	-	o	-
	P40089|P54999	v	-	-	-	-	o	-
	P40089|Q06406	v	-	-	-	-	o	-
	P34730|Q03497	v	-	o	-	-	-	-
	P05986|P41811	v	-	-	-	-	o	-
	P28495|P13517	v	-	-	-	-	o	-
	P53905|P40070	v	-	-	-	-	o	-
	P07276|Q00578	v	-	o	-	-	-	-
	P38622|P32485	v	-	-	-	-	o	o
	P16892|P23561	v	-	-	-	-	o	-
	P38182|P53867	v	-	-	-	-	o	o
	P22336|P26754	v	-	o	-	-	-	-
	P00546|P24583	v	o	-	-	-	-	o
	P32453|P00830	v	-	-	-	-	o	-
	P00830|P09457	v	-	-	-	o	-	-
	Q04226|P11747	v	-	o	-	-	o	-
	P19454|P15790	v	-	o	-	-	-	-
	P53141|P32492	v	-	-	-	-	o	-
	P36017|P39958	v	o	-	-	-	-	-
	P53845|P53039	v	-	-	-	-	o	-
	Q00772|Q01389	v	o	-	-	-	o	-
	P06787|P23287	v	-	o	-	-	o	-
	P05317|P10622	v	-	-	-	-	o	-
	Q07657|P25342	v	-	-	-	-	o	-

P34110|P38759	v	-	-	-	-	o	-
P25296|P23287	v	-	o	-	-	o	-
P40541|Q12158	v	-	-	-	-	-	o
P07251|P32453	v	-	-	-	-	o	-
P53905|P54999	v	-	-	-	-	o	-
Q99383|P38217	v	-	-	-	-	-	o
P10591|P25294	v	-	-	-	o	o	-
P38920|P14242	v	-	o	-	-	-	o
P25454|P06778	v	-	o	-	-	-	-
P32835|P41920	v	-	-	-	-	-	o
P32527|P38788	v	-	-	-	-	o	-
P00899|P00937	v	-	-	-	o	-	-
P21243|P23639	v	-	-	-	-	o	-
P15873|P38629	v	-	o	-	-	-	-
P47122|Q06543	v	-	-	-	-	o	o
P06243|P61864	v	-	-	-	-	o	-
P35191|P38523	v	-	-	-	o	-	-
P00546|P30283	v	o	o	-	-	o	-
P04821|P09435	v	-	-	-	-	o	-
Q54AF5|P09457	v	-	-	-	o	-	-
P32628|P61864	v	-	-	-	-	o	-
P32457|P25342	v	-	-	-	-	o	-
P19736|P32524	v	-	-	-	-	-	o
P53549|P20604	v	-	-	-	-	o	-
P32776|Q04673	v	-	o	-	-	-	-
P32854|P32602	v	-	-	-	-	o	-
P40343|P38753	v	-	-	-	-	o	-
P32485|P38623	v	-	-	-	-	o	o
Q03761|P53040	v	o	-	-	-	-	-
Q12004|Q00578	v	-	o	-	-	-	-
P38620|P32895	v	-	-	-	-	o	-
Q12280|P60010	v	-	-	-	-	o	-
P53131|P36048	v	-	-	-	-	o	-
P08018|P25390	v	o	o	-	-	-	-
P38630|P38251	v	-	-	-	-	o	o
P32776|P06839	v	-	o	-	-	-	-
P15108|P15705	v	-	-	-	-	o	o
Q02516|P06787	v	-	-	-	-	o	-
P05986|P07278	v	-	-	-	-	o	o
P15873|P32641	v	-	o	-	-	-	-
P36019|P06244	v	-	-	-	-	-	o
P06838|P06777	v	-	-	-	-	o	-
P38622|P16892	v	-	-	-	-	o	o
P06839|Q04673	v	-	o	-	-	-	-
P06245|P05986	v	-	o	-	-	-	-
P12398|P38523	v	-	-	-	o	-	-
P40054|P25382	v	-	-	-	-	o	-
P47017|P38203	v	-	-	-	-	o	-
P06245|P07278	v	-	-	-	-	o	o
P32864|P38146	v	o	-	-	-	-	-
P28274|Q08911	v	-	-	-	-	o	-
P07560|P39958	v	o	-	-	-	-	-
P29295|P34221	v	-	-	-	-	o	-
P14681|P06784	v	o	-	-	-	-	o
P40302|P23639	v	-	-	-	-	o	-
P38910|P19882	v	-	-	-	o	-	-
P26449|P41695	v	-	o	-	-	o	o
P40484|P53894	v	-	-	-	-	o	-
P33755|P53044	v	-	-	-	-	o	o
P32379|P21242	v	-	-	-	-	o	-

Q02336|P35177	v	-	-	-	-	o	-
Q12265|P38063	v	-	-	-	-	o	-
P28000|P16370	v	-	-	-	-	o	-
Q03048|P60010	v	-	-	-	-	o	-
P33298|P22204	v	-	-	-	-	-	o
Q06406|Q06217	v	-	-	-	-	o	-
P18898|P29703	v	-	-	-	-	o	-
P32835|P32499	v	-	-	-	-	-	o
P25491|P11484	v	-	-	-	o	o	-
Q07350|P19736	v	-	-	-	-	-	o
P19454|P38930	v	-	o	-	-	o	-
P38930|P43639	v	-	o	-	-	-	-
P21243|P23638	v	-	-	-	-	o	-
Q04673|Q12004	v	-	o	-	-	-	-
P02557|P09733	v	-	-	-	-	-	o
P32628|P33299	v	-	-	-	-	o	-
P15873|P38630	v	-	o	-	-	-	-
P60010|Q08873	v	-	-	-	-	o	-
P32628|P33297	v	-	-	-	-	o	-
Q05583|P38829	v	-	-	-	-	o	-
P32628|Q01939	v	-	-	-	-	o	-
P12398|P35191	v	-	-	-	o	o	-
P26754|P06778	v	-	o	-	-	-	-
P40348|P38251	v	-	-	-	-	o	o
P38555|P39958	v	o	-	-	-	-	-
P16474|P14906	v	-	-	-	-	o	-
P53112|P35056	v	-	-	-	-	o	-
P06780|P35688	v	-	o	-	-	-	-
P25454|P22336	v	-	o	-	-	-	-
P19454|P43639	v	-	o	-	-	o	-
P53905|Q06217	v	-	-	-	-	o	-
P22204|P40484	v	-	-	-	-	o	-
P32628|P40327	v	-	-	-	-	o	-
P36047|P32945	v	-	o	-	-	-	-
P33313|P47103	v	-	-	-	-	o	-
P40075|Q12451	v	-	-	-	-	o	-
P32628|Q02890	v	-	-	-	-	o	-
P32939|P05986	v	o	-	-	-	-	-
P26449|P47074	v	-	o	-	-	o	-
P53905|Q12330	v	-	-	-	-	o	-
P38146|P39958	v	o	-	-	-	-	-
Q12059|Q99344	v	-	-	-	-	o	-
P22138|P10964	v	-	-	-	o	-	-
P36019|P36018	v	-	-	-	-	-	o
P36149|Q03784	v	-	-	-	-	-	o
P00546|P53739	v	o	-	-	-	-	o
Q12309|P28004	v	-	-	-	-	o	-
P41808|P15705	v	-	-	-	-	-	o
P29703|P22007	v	-	-	-	-	o	-
P06787|P53141	v	-	o	-	-	o	-
P06774|P13434	v	o	-	-	-	-	-
P03965|P07258	v	-	-	-	o	-	-
P52286|P07834	v	-	-	-	-	o	o
Q06406|P53905	v	-	-	-	-	o	-
P38251|P32641	v	-	o	-	-	-	-
P38629|P38251	v	-	-	-	-	o	o
P39083|P19073	v	-	o	-	-	-	-
P02829|P23561	v	-	o	-	-	-	-
P22336|P06778	v	-	o	-	-	-	-
P19524|P06787	v	-	-	-	-	o	-

P29340|Q05568	v	-	-	-	-	o	-
P38930|P15790	v	-	o	-	-	o	-
P43563|P53894	v	-	-	-	-	o	-
P32458|P32468	v	-	-	-	-	o	-
Q12250|Q04062	v	-	-	-	-	o	-
P38920|P25847	v	-	-	-	o	-	-
P39940|P39003	v	-	-	-	-	o	-
P32485|P08018	v	o	-	-	-	-	o
P32776|Q12004	v	-	o	-	-	-	-
P16140|P17255	v	-	-	-	o	-	-
P38932|Q08144	v	-	-	-	-	o	-
P13434|Q02516	v	o	-	-	-	o	-
Q06217|Q12330	v	-	-	-	-	o	-
Q04493|P40005	v	-	-	-	-	-	o
P01123|P07560	v	o	-	-	-	-	o
P40339|P38251	v	-	-	-	-	o	o
Q00772|P32381	v	-	-	-	-	-	o
P32380|P53378	v	-	-	-	-	-	o
P16474|P25303	v	-	-	-	o	o	-
P40517|P32835	v	-	-	-	-	-	o
P00546|Q12263	v	-	-	-	-	-	o
P32447|Q6B1U3	v	-	-	-	-	o	-
P13382|P38121	v	-	-	-	-	o	-
P33298|P32381	v	-	-	-	-	o	-
P36017|P23561	v	o	-	-	-	-	-
Q03497|P19073	v	o	-	-	-	-	-
P06839|Q12004	v	-	o	-	-	-	-
P09032|P32501	v	-	-	-	-	o	-
P00830|Q54AF5	v	-	-	-	o	-	-
P36006|P06787	v	-	-	-	-	o	-
P32458|P32457	v	-	-	-	-	o	-
P32776|P07276	v	-	o	-	-	-	-
Q08951|P47064	v	-	-	-	-	o	-
P38620|Q12265	v	-	-	-	-	o	-
P43639|P15790	v	-	o	-	-	o	-
P53141|P08964	v	-	-	-	-	o	-
P32838|Q12199	v	-	-	-	-	o	-
Q03940|Q12464	v	-	-	-	-	o	-
P40089|Q06217	v	-	-	-	-	o	-
P16892|P06784	v	o	-	-	-	-	o
P40348|P38629	v	-	-	-	-	o	o
P52286|P39014	v	-	-	-	-	o	o
P06787|P32492	v	-	-	-	-	o	-
Q04439|P06787	v	-	-	-	-	o	-
Q03919|P52491	v	-	o	-	-	-	-
P46948|Q05636	v	-	-	-	-	o	-
P38630|P40339	v	-	-	-	-	o	o
P21243|P40303	v	-	-	-	-	o	-
P20604|P39960	v	-	-	-	-	-	o
P15873|P40339	v	-	o	-	-	-	-
Q12469|P19073	v	o	-	-	-	-	-
Q06217|P40070	v	-	-	-	-	o	-
P40089|P53905	v	-	-	-	-	o	-
P25342|P32468	v	-	-	-	-	o	-
P38217|P32835	v	-	-	-	-	o	-
P36018|P39958	v	o	-	-	-	-	-
P40089|P40070	v	-	-	-	-	o	-
Q99383|P25299	v	-	-	-	-	o	-
P00830|Q96101	v	-	-	-	o	-	-
P48562|P19073	v	o	-	-	-	-	-

Q99189|P32835	v	-	-	-	-	-	o
P28000|P07703	v	-	-	-	-	o	-
P21242|P21243	v	-	-	-	-	o	-
P20459|P32481	v	-	-	-	-	o	-
P32939|P39958	v	o	-	-	-	-	-
Q07844|Q01939	v	-	-	-	-	o	-
Q06406|P40070	v	-	-	-	-	o	-
P06244|P07278	v	-	-	-	-	o	o
P25491|P10591	v	-	-	-	o	o	-
P40484|P32328	v	-	-	-	-	o	-
P06774|Q02516	v	o	-	-	-	o	-
P32836|P32835	v	-	o	-	-	-	-
P33331|P32835	v	-	o	-	-	-	-
Q06142|Q02821	v	-	o	-	-	-	o
P00546|P24870	v	o	o	-	-	-	-
Q00916|P08964	v	-	-	-	-	o	-
P09457|Q96101	v	-	-	-	o	-	-
P33298|P25454	v	-	-	-	-	o	-
P05317|P05318	v	-	-	-	-	o	-
P36019|P32864	v	o	-	-	-	-	-
P39010|P18851	v	-	-	-	-	o	-
P32473|P16387	v	-	o	-	-	o	o
P15873|P38251	v	-	o	-	-	-	-
P14741|P12754	v	-	-	-	-	o	-
P38879|P40314	v	-	-	-	-	o	o
P14680|P50101	v	-	-	-	-	o	-
P61864|P14682	v	-	o	-	-	-	-
P43321|P36048	v	-	-	-	-	o	-
P52488|Q06624	v	-	-	-	-	o	-
P32457|Q07657	v	-	-	-	-	o	-
P02829|P15705	v	-	-	-	-	o	o
P32485|P23561	v	o	-	-	-	o	-
P04051|P22276	v	-	-	-	o	-	-
P06838|P28519	v	-	o	-	-	o	-
P25847|P14242	v	-	-	-	o	-	-
P33297|P22204	v	-	-	-	-	-	o
P10591|P04821	v	-	-	-	-	o	-
P32458|Q04921	v	-	-	-	-	o	-
P22336|P38111	v	-	o	-	-	-	-
P40096|Q92317	v	-	-	-	-	o	-
P32773|P32774	v	-	-	-	-	o	-
P00546|P24869	v	o	o	-	-	-	-
P60010|P07274	v	-	-	-	-	o	-
P40348|P32641	v	-	o	-	-	-	-
Q04673|Q00578	v	-	o	-	-	-	-
P25299|P04147	v	-	-	-	-	o	o
Q12330|P54999	v	-	-	-	-	o	-
P00546|P23337	v	-	-	-	-	-	o
P00546|P24868	v	o	o	-	-	-	-
P28519|P06777	v	-	-	-	-	o	-
P40302|P32496	v	-	-	-	-	o	-
P33313|P15108	v	-	-	-	-	o	-
P32598|P36047	v	-	o	-	-	-	-
P32864|P36017	v	o	-	-	-	-	-
P07251|P09457	v	-	-	-	o	-	-
Q99260|P39958	v	o	-	-	-	-	-
Q12018|P52286	v	-	o	-	-	-	-
P11747|Q12030	v	-	o	-	-	-	-
P32497|P32911	v	-	-	-	-	o	-
P00546|P40480	v	-	o	-	-	-	-

Q12199|P23594	v	-	-	-	-	o	-
Q07657|P32468	v	-	-	-	-	o	-
P89886|P47089	v	-	-	-	-	o	-
P38339|Q00246	v	-	o	-	-	-	-
P40348|P40339	v	-	-	-	-	o	o
Q04493|P52553	v	-	-	-	-	o	-
Q12488|Q03406	v	-	-	-	-	o	-
Q01389|P32490	v	o	o	-	-	-	-
P40036|P26570	v	-	-	-	-	o	-
P20133|Q00618	v	-	-	-	-	o	-
P06839|Q00578	v	-	o	-	-	-	-
P47083|P32899	v	-	-	-	-	o	-
P38624|P41808	v	-	-	-	-	o	-
P20081|P32600	v	-	o	-	-	-	-
P51996|P39958	v	o	-	-	-	-	-
P07251|Q54AF5	v	-	-	-	o	-	-
Q03654|P28004	v	-	-	-	-	o	-
P60010|P17555	v	-	-	-	-	o	-
P18562|P27515	v	-	-	-	-	o	-
P24783|P05736	v	-	-	o	-	-	o
Q05027|P53040	v	o	-	-	-	-	-
P53691|P05986	v	-	o	-	-	-	-
P10622|P02400	v	-	-	-	-	-	o
P32502|P12754	v	-	-	-	-	o	-
Q92331|P05453	v	-	-	-	-	o	-
P06244|P06245	v	-	o	-	-	-	-
Q00772|P38692	v	o	-	-	-	-	o
P32491|Q01389	v	o	o	-	-	-	-
P06787|P14747	v	-	o	-	-	o	-
P35056|P13711	v	-	-	-	-	o	-
P23639|P23638	v	-	-	-	-	o	-
P16370|P38902	v	-	-	-	-	o	-
P36019|P36017	v	o	-	-	-	-	o
P00359|P06700	v	-	-	-	-	o	-
P32836|P33331	v	-	o	-	-	-	-
P61864|P48510	v	-	-	-	-	-	o
P38629|P40339	v	-	-	-	-	o	o
P14907|P48837	v	-	-	-	-	o	-
P01123|P39958	v	o	-	-	-	-	-
P36018|P36017	v	o	-	-	-	-	o
P52286|P24814	v	-	-	-	-	o	-
P25655|P31384	v	-	-	-	-	o	-
P21672|P21524	v	-	-	-	o	-	-
P19524|P53141	v	-	-	-	-	o	-
Q12199|P32345	v	-	-	-	-	o	-
P01123|P32864	v	o	-	-	-	-	-
P06780|P39083	v	-	o	-	-	-	-
P32776|Q00578	v	-	o	-	-	-	-
Q06217|P54999	v	-	-	-	-	o	o
P27466|P06787	v	-	-	-	-	o	-
Q04491|P38968	v	-	-	-	-	o	-
P38920|Q07980	v	-	o	-	-	-	o
P06839|P07276	v	-	o	-	-	-	-
P43588|Q08723	v	-	-	-	-	o	-
Q00539|Q03776	v	-	-	-	-	o	-
Q00539|P39682	v	-	-	-	-	o	-
P08018|P53599	v	o	o	-	-	-	-
P47122|Q08726	v	-	-	-	-	o	o
P35056|P32796	v	-	-	-	-	o	-
Q02554|Q99181	v	-	-	-	-	o	-

	P32602|Q08144	v	-	-	-	-	o	-
	P38689|P32895	v	-	-	-	-	o	-
	P14681|P23561	v	-	-	-	-	o	-
	Q06102|P45976	v	-	-	-	-	o	-
	Q03654|Q12309	v	-	-	-	-	o	-
	P15873|P40348	v	-	o	-	-	-	-
	P25298|P25299	v	-	-	-	-	o	-
	P33307|Q02821	v	-	-	-	-	o	-
M.musculus	Q52L79|Q61328	-	v	o	-	-	-	-
	Q4FJM2|P43063	-	v	o	-	-	-	-
	P06400|420232	-	v	o	-	-	-	-
	Q13873|P27040	-	v	o	-	-	-	-
	P63001|Q61036	o	v	-	-	-	-	-
	P15056|Q63932	o	v	-	-	-	-	-
	Q5SQP3|Q5I0Y2	-	v	o	-	-	-	-
	O35608|Q02858	-	v	o	-	-	-	-
	P97369|Q09014	-	v	o	-	-	-	-
	7274392|Q548P0	-	v	o	-	-	-	-
	P01900|P01887	-	v	o	-	-	-	-
	P01887|Q792Z7	-	v	o	-	-	-	-
	P08476|P27038	-	v	o	-	-	-	-
	P12644|P37172	-	v	o	-	-	-	-
	Q13873|Q53Z43	-	v	o	-	-	-	-
	P12644|Q53Z43	-	v	o	-	-	-	-
	P36898|P22004	-	v	o	-	-	-	-
	P35279|Q541Z9	o	v	-	-	-	-	-
	Q548Y4|P25799	-	v	o	-	-	o	-
	P27037|Q53Z43	-	v	o	-	-	-	-
	P36898|P27037	-	v	o	-	-	-	-
	P25916|2143517	-	v	-	-	-	o	-
	P36898|P27040	-	v	o	-	-	-	-
	P30285|Q790L7	o	v	o	-	-	o	-
	P63139|Q62725	o	v	-	-	-	-	-
	7274392|Q5QNW2	-	v	o	-	-	-	-
	Q13873|P37172	-	v	o	-	-	-	-
	P61019|P20339	o	v	-	-	-	-	-
	P15056|P31938	o	v	-	-	-	-	-
	109791|P15923	-	v	o	-	-	-	-
	P03372|Q9JLP2	-	v	o	-	-	-	-
	P54130|P16092	-	v	o	-	-	-	-
	P27037|P37172	-	v	o	-	-	-	-
	P01101|Q569U6	-	v	o	-	-	-	-
	P27037|P12644	-	v	o	-	-	-	-
	O43927|Q04683	-	v	o	-	-	-	-
	P47873|Q64385	-	v	o	-	-	-	-
	P27040|P18075	-	v	o	-	-	-	-
	Q53Z43|P27040	-	v	o	-	-	-	-
	P23150|P06343	-	v	o	-	-	-	-
	P13346|Q52L79	-	v	o	-	-	-	-
	P97377|Q564P6	-	v	o	-	-	-	-
	P01108|110731	-	v	-	-	-	o	-
	P37172|P18075	-	v	o	-	-	-	-
	O70441|P60879	-	v	-	-	-	o	-
	Q5NCK7|1083499	o	v	-	-	-	-	o
	P63139|P23708	o	v	-	-	-	-	-
	Q4FK45|Q564P6	-	v	o	-	-	-	-
	Q61851|P54130	-	v	o	-	-	-	-
	P37237|Q61851	-	v	o	-	-	-	-
	P37172|P43026	-	v	o	-	-	-	-
	P36941|Q9QYH9	-	v	o	-	-	-	-

Q6HAA1|Q00420	-	v	-	-	-	o	-
P27037|P43026	-	v	o	-	-	-	-
Q02858|O08538	-	v	o	-	-	-	-
P43488|P47741	-	v	o	-	-	-	-
P36898|P12644	-	v	o	-	-	-	-
P01887|P11609	-	v	o	-	-	-	-
P27037|P27040	-	v	o	-	-	-	-
P18265|Q6GSA6	o	v	-	-	-	-	o
423499|1083499	o	v	o	-	-	-	-
P37172|P27040	-	v	o	-	-	-	-
631889|P60879	-	v	-	-	-	o	-
P61019|Q541Z9	o	v	-	-	-	-	-
P10085|P15923	-	v	o	-	-	-	-
Q13873|P22004	-	v	o	-	-	-	-
P36898|P43026	-	v	o	-	-	-	-
Q53Z43|P43026	-	v	o	-	-	-	-
P97377|Q790L7	o	v	o	-	-	o	-
P22004|P37172	-	v	o	-	-	-	-
P35279|P61019	o	v	-	-	-	-	-
Q53Z43|P18075	-	v	o	-	-	-	-
P62821|P61019	o	v	-	-	-	-	-
Q64261|Q4FK45	-	v	o	-	-	-	-
P27037|P18075	-	v	o	-	-	-	-
P12644|P27040	-	v	o	-	-	-	-
Q545V0|Q61036	o	v	-	-	-	-	-
P30285|Q5FWJ6	-	v	-	-	-	o	-
P01111|110873	o	v	-	-	-	-	-
P01101|Q52L79	-	v	o	-	-	-	-
Q5HZK4|Q60996	-	v	-	-	-	o	-
Q53Z43|P22004	-	v	o	-	-	-	-
P22004|P27040	-	v	o	-	-	-	-
P13346|Q569U6	-	v	o	-	-	-	-
P37237|P16092	-	v	o	-	-	-	-
O15520|P21803	-	v	o	-	-	-	-
Q541Z9|P20339	o	v	-	-	-	-	-
P32851|P60879	-	v	-	-	-	o	-
P62821|P20339	o	v	-	-	-	-	-
Q5SPX6|O88821	-	v	o	-	-	-	-
P27040|P43026	-	v	o	-	-	-	-
P01887|Q7JJ15	-	v	o	-	-	-	-
P30285|Q564P6	-	v	o	-	-	-	-
Q63953|110873	-	v	o	-	-	-	-
P36898|Q13873	-	v	o	-	-	-	-
P70304|Q03145	-	v	-	-	-	o	o
P41274|P20334	-	v	o	-	-	-	-
P19878|P97369	-	v	o	-	-	-	-
1363982|Q16514	o	v	-	-	-	-	-
Q13873|P12644	-	v	o	-	-	-	-
P20181|P15209	-	v	o	-	-	-	-
Q5NC86|Q60996	-	v	-	-	-	o	-
Q64261|Q564P6	-	v	o	-	-	-	-
Q564P6|Q790L7	-	v	o	-	-	-	-
P97377|Q4FK45	-	v	o	-	-	-	-
Q16594|1363982	o	v	-	-	-	-	-
P36898|P18075	-	v	o	-	-	-	-
Q13873|P43026	-	v	o	-	-	-	-
Q01094|420232	-	v	o	-	-	o	-
P09759|Q60631	-	v	-	-	-	o	-
P15209|Q541P3	-	v	o	-	-	-	-
Q13873|P18075	-	v	o	-	-	-	-

	P23708|Q62725	o	v	-	-	-	o	-
	P35279|P20339	o	v	-	-	-	-	-
	Q64261|Q790L7	o	v	o	-	-	o	-
	P39428|P39429	-	v	o	-	-	-	-
	P62821|Q541Z9	o	v	-	-	-	-	-
	423499|Q5NCK7	o	v	-	-	-	-	-
	Q08850|P60879	-	v	-	-	-	o	-
	P30561|2119731	-	v	o	-	-	-	-
	P27037|P22004	-	v	o	-	-	-	-
	P39429|P20333	-	v	o	-	-	-	-
	P62821|P35279	o	v	-	-	-	-	-
	Q01094|P06400	-	v	o	-	-	-	-
	P39688|602251	-	v	-	-	-	o	-
	Q00560|Q5SPX6	-	v	o	-	-	-	-
	P01101|Q61328	-	v	o	-	-	-	-
	P30285|Q4FK45	-	v	o	-	-	-	-
H.sapiens	P09038|P11362	-	o	v	-	-	-	-
	Q6FI05|P06493	-	o	v	-	-	-	-
	P13862|Q4VX47	o	-	v	-	-	-	-
	Q5T1U9|P07992	o	-	v	-	-	o	-
	P06730|Q62622	-	-	v	-	-	o	-
	P25942|Q13114	-	o	v	-	-	-	-
	Q6FI00|Q00534	-	o	v	-	-	-	-
	Q567U5|Q6FH52	o	-	v	-	-	-	-
	Q5VXF1|Q53S29	o	-	v	-	-	-	-
	Q14974|P52292	o	-	v	-	-	-	-
	Q53XN3|Q6IBH7	-	-	v	-	-	o	-
	P61968|O60617	-	-	v	-	-	o	-
	P61769|Q5HYM5	-	o	v	-	-	-	-
	Q04917|P31946	o	-	v	-	-	-	-
	P19838|P19838	-	o	v	-	-	o	-
	Q6FH50|Q16288	-	o	v	-	-	-	-
	P15173|105866	-	o	v	-	-	-	-
	Q5U035|P46527	-	o	v	-	-	-	-
	P12004|O75943	o	-	v	-	-	-	-
	O75943|Q6FHX7	o	-	v	-	-	-	-
	Q6FI00|Q6FI05	-	o	v	-	-	-	-
	P46527|P11802	-	o	v	-	-	-	-
	Q5HYA0|Q02763	-	o	v	-	-	-	-
	Q53S51|P51684	-	o	v	-	-	-	-
	P01137|Q5T7S2	-	o	v	-	-	-	-
	Q6FI00|P46527	-	o	v	-	-	-	-
	Q99759|Q567U5	o	-	v	-	-	-	-
	P18074|P28715	o	-	v	-	-	-	-
	P21802|P11487	-	o	v	-	-	-	-
	P13862|P68400	o	-	v	-	-	o	-
	Q6FI00|P06493	o	o	v	-	-	o	-
	P19838|Q15653	-	-	v	-	-	o	-
	Q5JSB4|Q15329	-	o	v	-	-	o	-
	Q6I9Y7|P28715	o	-	v	-	-	-	-
	P24941|Q5U035	-	o	v	-	-	-	-
	P27694|Q6FHX9	o	-	v	-	-	-	-
	Q04206|P19838	-	o	v	-	-	o	-
	P63208|Q13616	o	-	v	-	-	-	-
	P28715|Q13888	o	-	v	-	-	-	-
	P20248|Q00534	-	o	v	-	-	-	-
	P05412|Q6ICQ9	-	o	v	-	-	-	-
	Q6FGE9|Q00534	o	-	v	-	-	-	-
	Q6ICR7|P32248	-	o	v	-	-	-	-
	P25963|Q04864	-	-	v	-	-	o	-

P37023|P37173	-	o	v	-	-	-	-
P22607|P08620	-	o	v	-	-	-	-
P19838|Q01201	-	o	v	-	-	o	-
Q6FH50|Q91407	-	o	v	-	-	-	-
Q04206|Q15653	-	-	v	-	-	o	-
P28749|Q5JSB4	-	o	v	-	-	-	-
P15884|Q02535	-	o	v	-	-	-	-
Q30201|P61769	-	o	v	-	-	-	-
P05230|P22607	-	o	v	-	-	-	-
P40692|P54278	o	-	v	-	-	-	o
Q5STB3|339760	-	o	v	-	-	-	-
Q6FG41|P05412	-	o	v	-	-	-	-
Q04917|P62258	o	-	v	-	-	-	-
P01138|Q01973	-	o	v	-	-	-	-
Q6FI05|P20248	-	o	v	-	-	-	-
O00221|P19838	-	-	v	-	-	o	-
Q01201|P19838	-	o	v	-	-	o	-
Q658U1|Q5T1L5	-	o	v	-	-	-	-
Q598Q1|Q5VVP4	-	o	v	-	-	-	-
Q5T7S2|Q4VAV9	-	o	v	-	-	-	-
Q99759|P31946	o	-	v	-	-	-	-
P24941|P38936	-	o	v	-	-	-	-
Q6I9Y7|Q13888	o	-	v	-	-	-	-
P18074|Q13889	o	-	v	-	-	-	-
P06493|P46527	-	o	v	-	-	-	-
P24864|Q6FI05	-	o	v	-	-	-	-
P21802|Q6FGV5	-	o	v	-	-	-	-
Q15653|P19838	-	-	v	-	-	o	-
Q04206|Q01201	-	o	v	-	-	-	-
P62258|P31946	o	-	v	-	-	-	-
P19784|Q4VX47	o	-	v	-	-	-	-
O15111|Q5TGM6	o	-	v	-	-	-	-
Q5JSB4|Q16254	-	o	v	-	-	o	-
P38936|P20248	-	o	v	-	-	-	-
Q01094|Q14188	-	o	v	-	-	o	-
P12034|P11362	-	o	v	-	-	-	-
P17080|P61970	o	-	v	-	-	-	-
P10600|P37173	-	o	v	-	-	-	-
P25963|Q04206	-	-	v	-	-	o	-
P10600|P37023	-	o	v	-	-	-	-
Q5TER1|Q4VC47	o	-	v	-	-	-	-
Q00653|Q04864	-	o	v	-	-	o	-
Q14209|Q14188	-	o	v	-	-	o	-
P12643|882391	-	o	v	-	-	-	-
P42702|Q6FHP8	-	o	v	-	-	-	-
Q6I9Y7|Q53QM0	o	-	v	-	-	-	-
Q6FI00|P38936	-	o	v	-	-	-	-
Q13888|Q13889	o	-	v	-	-	-	-
Q04917|Q6FH52	o	-	v	-	-	-	-
P05230|P22455	-	o	v	-	-	-	-
P43351|Q6FHX9	o	-	v	-	-	-	-
Q5U035|Q00534	-	o	v	-	-	-	-
Q5JSB4|Q01094	-	o	v	-	-	o	-
Q13888|Q53QM0	o	-	v	-	-	-	-
Q99759|Q6FH52	o	-	v	-	-	-	-
P05412|P18848	-	o	v	-	-	-	-
P22455|O95750	-	o	v	-	-	-	-
O15111|O14920	o	-	v	-	-	-	-
Q04206|O00221	-	-	v	-	-	o	-
Q5U035|P38936	-	o	v	-	-	-	-

P18074|Q53QM0	o	-	v	-	-	-	-
Q6ICQ9|P05412	-	o	v	-	-	-	-
Q04206|Q00653	-	o	v	-	-	o	-
Q6IAZ1|Q5Y7D1	-	o	v	-	-	-	-
Q15435|P62136	o	-	v	-	-	-	-
P24941|P20248	o	o	v	-	-	o	-
P26927|Q04912	-	-	v	-	-	o	-
P05230|P21802	-	o	v	-	-	-	-
P46527|P20248	-	o	v	-	-	-	-
P14598|P19878	-	o	v	-	-	-	-
P46527|Q00534	-	o	v	-	-	-	-
P32248|Q6IBD6	-	o	v	-	-	-	-
Q00653|Q00653	-	o	v	-	-	o	-
Q13233|P07900	o	-	v	-	-	-	-
P38936|P11802	-	o	v	-	-	-	-
P63208|995826	-	-	v	-	-	o	-
Q5U035|P11802	o	o	v	-	-	o	-
P29597|P17181	-	o	v	-	-	-	-
P19838|Q04206	-	o	v	-	-	o	-
Q6IAZ1|P04229	-	o	v	-	-	-	-
Q99759|Q04917	o	-	v	-	-	-	-
Q5TK76|P61769	-	o	v	-	-	-	-
O00221|Q00653	-	-	v	-	-	o	-
Q16254|Q14188	-	o	v	-	-	o	-
P09038|P22455	-	o	v	-	-	-	-
O00221|Q04864	-	-	v	-	-	o	-
Q5CAQ6|P35869	-	o	v	-	-	-	-
P28715|Q53QM0	o	-	v	-	-	-	-
P22607|P12034	-	o	v	-	-	-	-
Q00653|Q15653	-	-	v	-	-	o	-
Q567U5|P31946	o	-	v	-	-	-	-
P06493|P14635	o	o	v	-	-	o	-
P81274|P04899	-	-	v	-	-	o	o
Q5JSB4|Q14209	-	o	v	-	-	o	-
P61769|Q542Z3	-	o	v	-	-	-	-
Q01201|Q00653	-	o	v	-	-	o	-
P41273|Q07011	-	o	v	-	-	-	-
P21802|P31371	-	o	v	-	-	-	-
Q5STB3|P36941	-	o	v	-	-	-	-
Q04206|Q04864	-	o	v	-	-	-	-
P21802|P09038	-	o	v	-	-	-	-
Q567U5|P62258	o	-	v	-	-	-	-
P78527|P27694	o	-	v	-	-	-	-
Q07960|P60953	o	-	v	-	-	-	-
P49459|P62988	o	-	v	-	-	-	-
P36873|P41236	-	-	v	-	-	o	o
Q6FH52|P62258	o	-	v	-	-	-	-
Q15544|Q5TYV6	o	-	v	-	-	o	-
O08816|P16333	-	-	v	-	-	o	-
P01138|P04629	-	o	v	-	-	-	-
P61769|Q546I9	-	o	v	-	-	-	-
P20809|Q5VZ79	-	o	v	-	-	-	-
O95407|Q6FHA1	-	o	v	-	-	-	-
Q02763|O15123	-	o	v	-	-	-	-
Q12962|Q5TYV6	o	-	v	-	-	-	-
P59773|P26368	-	-	v	-	-	o	o
P12643|Q13873	-	o	v	-	-	-	-
P24941|Q6FI05	-	o	v	-	-	-	-
P19838|O00221	-	-	v	-	-	o	-
Q00653|P19838	-	o	v	-	-	o	-

P17080|P62826	o	-	v	-	-	-	-
P01137|P37173	-	o	v	-	-	-	-
Q13616|P62877	o	-	v	-	-	-	-
Q6FGV5|P21802	-	o	v	-	-	-	-
Q52LZ2|P42702	-	o	v	-	-	-	-
P24864|P06493	o	o	v	-	-	o	-
P06493|P38936	-	o	v	-	-	-	-
P01137|P37023	-	o	v	-	-	-	-
P61769|Q6DU50	-	o	v	-	-	-	-
Q5T7S2|P10600	-	o	v	-	-	-	-
P61970|P62826	o	-	v	-	-	-	-
Q15653|Q04864	-	-	v	-	-	o	-
Q6I9Y7|Q13889	o	-	v	-	-	-	-
P13862|P19784	o	-	v	-	-	o	-
P27694|Q13156	o	-	v	-	-	-	-
P09038|P22607	-	o	v	-	-	-	-
Q6FI00|P24941	o	o	v	-	-	o	-
Q15653|Q00653	-	-	v	-	-	o	-
P25963|Q00653	-	-	v	-	-	o	-
P19838|Q00653	-	o	v	-	-	o	-
Q6FI00|P11802	-	o	v	-	-	-	-
P05230|P11362	-	o	v	-	-	-	-
P20248|P11802	-	o	v	-	-	-	-
P25942|Q5T1L5	-	o	v	-	-	-	-
P35739|Q598Q1	-	o	v	-	-	-	-
P68400|Q4VX47	o	-	v	-	-	o	-
P06493|P20248	o	o	v	-	-	o	-
Q53ZZ1|O95407	-	o	v	-	-	-	-
P01138|P35739	-	o	v	-	-	-	-
P12694|Q9BQL0	o	-	v	-	-	o	o
P25963|P19838	-	-	v	-	-	o	-
Q6FH52|P31946	o	-	v	-	-	-	-
Q6FGF6|Q53R18	-	o	v	-	-	-	-
P63208|P62877	o	-	v	-	-	-	-
P61769|Q53Z42	-	o	v	-	-	-	-
P19838|Q04864	-	o	v	-	-	o	-
P24941|P46527	-	o	v	-	-	-	-
P18848|Q6ICQ9	-	o	v	-	-	-	-
P61769|Q5SS57	-	o	v	-	-	-	-
P12004|Q6FHX7	o	-	v	-	-	-	-
P18074|Q13888	o	-	v	-	-	-	-
Q5JZA5|Q5T7M0	-	o	v	-	-	-	-
Q99759|P62258	o	-	v	-	-	-	-
P28749|Q14188	-	o	v	-	-	-	-
Q6FGV5|P22607	-	o	v	-	-	-	-
Q04756|P08581	-	-	v	-	-	o	-
P28749|Q16254	-	o	v	-	-	-	-
Q99759|P07900	o	-	v	-	-	-	-
Q5T7S2|P37173	-	o	v	-	-	-	-
P61769|P03989	-	o	v	-	-	-	-
P13501|P49682	-	o	v	-	-	-	-
Q5JSB4|O00716	-	o	v	-	-	o	-
Q6FGF6|Q53H00	-	o	v	-	-	-	-
Q5STV3|P36941	-	o	v	-	-	-	-
Q13889|Q53QM0	o	-	v	-	-	-	-
Q00653|Q01201	-	o	v	-	-	o	-
Q08209|Q53SL0	o	-	v	-	-	o	-
Q13156|P43351	o	-	v	-	-	-	-
O60566|O43684	o	-	v	-	-	o	o
P19784|P68400	o	-	v	-	-	-	-

	Q28147|P36873	-	-	v	-	-	o	-
	Q04917|Q567U5	o	-	v	-	-	-	-
	19909966|P63208	-	-	v	-	-	o	-
	Q99759|Q13163	o	o	v	-	-	-	-
	P42768|P16333	-	-	v	-	-	o	-
	P21802|P10767	-	o	v	-	-	-	-
	P27694|P43351	o	-	v	-	-	-	-
	Q92731|Q13105	-	o	v	-	-	-	-
	P23458|P17181	-	o	v	-	-	-	-
	Q6FG41|P18848	-	o	v	-	-	-	-
	P18074|Q6I9Y7	o	-	v	-	-	-	-
H.pylori	O25153|P71403	-	-	-	v	o	-	-
	O25629|P56097	-	-	-	v	o	-	-
	P56001|O25676	-	-	-	v	o	-	-
	P55993|O25806	-	-	-	v	o	-	-
	O25806|O25676	-	-	-	v	o	-	-
	P55993|P56001	-	-	-	v	o	-	-
	O25029|P56030	o	-	-	v	-	-	o
	O25806|P56001	-	-	-	v	o	-	-
E.coli	P00822|P00831	o	-	-	-	v	-	-
	P0A8T7|P00579	-	-	-	o	v	-	-
	P0A6N1|P0A6P1	-	-	-	-	v	o	-
	P0A6F5|P0A9S3	-	-	-	-	v	o	-
	P00831|P00837	o	-	-	-	v	-	-
	P0A6F5|P0A9B2	-	-	-	-	v	o	-
	P00824|P00831	o	-	-	-	v	-	-
	P00822|P00837	o	-	-	-	v	-	-
	P00831|P00855	o	-	-	-	v	-	-
	Q547V3|P23909	o	-	-	-	v	-	-
	P00822|P00855	o	-	-	-	v	-	-
	P0A7Z4|P31804	-	-	-	o	v	-	-
	P0A6Y8|P08622	o	-	-	-	v	o	-
	P39452|P00452	o	-	-	-	v	-	-
	P0A9A6|P06137	-	-	-	o	v	-	-
	P00824|P00837	o	-	-	-	v	-	-
	P00824|P00855	o	-	-	-	v	-	-
	P0A6F5|P0A6F9	o	-	-	-	v	-	-
	P00968|P0A6F1	o	-	-	-	v	-	-
	P0A7Z4|P0A8V2	-	-	-	o	v	-	-
	P00822|P00824	o	-	-	-	v	-	-
	P00579|P0A8V2	-	-	-	o	v	-	-
	P09372|P08622	o	-	-	-	v	-	-
	P06143|P07363	-	-	-	o	v	-	-
	P0A6F5|P07913	-	-	-	-	v	o	-
	P00579|P0A7Z4	-	-	-	o	v	-	-
	P0A8T7|P0A7Z4	-	-	-	o	v	-	-
	P00903|P05041	o	-	-	-	v	-	-
	P0A8V2|P31804	-	-	-	o	v	-	-
	P09372|P0A6Y8	o	-	-	-	v	-	-
	P06143|P22763	-	-	-	o	v	-	-
	P0A8T7|P31804	-	-	-	o	v	-	-
	P0A8T7|P0A8V2	o	-	-	-	v	-	-
D.melanogaster	Q9VXP1|P52486	o	-	-	-	-	v	-
	Q9W3V9|Q9VSY9	o	o	-	-	-	v	-
	O46085|Q9VB02	o	-	-	-	-	v	-
	Q03043|O18640	o	-	-	-	-	v	-
	Q9VPT8|Q9VN31	o	-	-	-	-	v	-
	Q9VLV5|Q9VI10	o	-	-	-	-	v	-
	P91632|Q8INP0	-	-	-	-	-	v	o
	24649768|P62152	-	-	-	-	-	v	o

Q9V7D3|Q9W541	o	-	-	-	-	v	-
Q9W3N6|Q7KU23	-	-	-	-	-	v	o
Q9VMV0|Q9VCA8	-	-	o	-	-	v	-
O77430|Q7YZ95	-	-	o	-	-	v	-
Q9VLV5|Q9VJI7	o	-	-	-	-	v	-
Q9V830|24646318	o	-	-	-	-	v	-
Q9W424|Q9VQK7	o	-	-	-	-	v	-
Q9VHA8|24646318	o	-	-	-	o	v	-
24666323|Q95NR0	-	-	-	-	-	v	o
Q9W1S2|P13709	o	-	-	-	-	v	-
Q9V754|Q9VUZ1	o	-	-	-	-	v	-
Q9VL21|O18353	-	-	o	-	-	v	-
Q9VRT7|24583408	o	-	-	-	-	v	-
O77430|Q9W214	o	-	-	-	-	v	-
Q8IQN4|Q9VU34	-	-	-	-	-	v	o
Q8IP90|Q8IMS8	-	-	-	-	-	v	o
Q541C2|Q9VK34	o	-	-	-	-	v	-
Q9VM49|Q9W424	o	-	-	-	-	v	-
Q8MQK4|Q9VRQ1	o	-	-	-	-	v	-
O97183|Q9VJE4	o	-	-	-	-	v	-
P48598|Q9XZ56	-	-	o	-	-	v	-
Q9Y0X3|P28518	o	-	o	-	-	v	-
Q9V436|Q9VK14	o	-	-	-	-	v	-
Q9U1I1|Q8INK4	-	-	-	-	-	v	o
Q540V5|P54359	o	-	-	-	-	v	-
Q9V780|24652112	o	-	-	-	-	v	-
Q9V7P0|Q9V6B9	o	-	-	-	-	v	-
P09089|P42003	-	-	-	-	-	v	o
Q24318|Q5U0Z6	-	o	o	-	-	v	-
Q9W4H8|Q7KRY6	o	-	-	-	-	v	-
Q86B59|Q9VCF4	o	-	-	-	-	v	-
Q9VEN1|Q9W579	-	-	-	-	-	v	o
Q9VJ00|O18353	-	-	o	-	-	v	-
P08570|P19889	o	-	-	-	-	v	-
P16378|Q9VB22	-	-	o	-	-	v	o
P49028|Q9V535	-	-	-	-	-	v	o
O46106|Q9VEP9	-	-	-	-	-	v	o
Q9VIF7|Q9VAT0	-	-	o	-	-	v	-
Q9V3C7|Q4PIY5	-	-	o	-	-	v	o
Q9VHA8|Q5BI92	o	-	-	-	o	v	-
Q8ST61|Q9W256	o	-	-	-	-	v	-
P52295|Q9VMQ0	-	-	-	-	-	v	o
P52295|24584736	o	-	-	-	-	v	-
Q9V7W9|Q5U0Y2	-	-	o	-	-	v	-
Q9VU67|Q9V3R3	o	-	-	-	-	v	o
P16914|Q9VYI0	o	-	-	-	-	v	o
Q5U0U0|Q9VWR1	o	-	-	-	-	v	-
O18413|O77135	o	-	-	-	-	v	-
Q53YH7|24648319	-	-	-	-	o	v	-
Q9V3H2|P26270	o	-	-	-	-	v	-
Q9VCD5|Q8IGR0	o	-	-	-	-	v	-
Q7KVY7|Q9VH76	-	o	-	-	-	v	-
Q9VWC4|Q9VF10	o	-	o	-	-	v	-
P45594|P83967	o	-	-	-	-	v	-
Q9VK46|Q8IMR4	o	-	-	-	-	v	-
Q9W1T4|Q94535	-	-	o	-	-	v	o
Q7KR26|Q9VAH0	-	-	o	-	-	v	-
Q4V6Y5|Q9VYS4	-	-	-	-	-	v	o
Q9VLV5|Q24297	o	-	-	-	-	v	-
Q9VJI7|Q9VI10	o	-	-	-	-	v	-

Q9VHA8|Q9BIR7	o	-	-	-	o	v	-
Q24117|Q9W2J4	-	-	-	-	-	v	o
P52654|P52656	o	-	-	-	-	v	-
P84249|Q9V464	o	-	-	-	-	v	-
Q5U0U0|P45594	o	-	-	-	-	v	-
Q86R94|Q9VEQ2	-	-	-	-	-	v	o
Q9V472|O18353	-	-	o	-	-	v	-
Q94518|Q8IR50	-	-	-	-	-	v	o
Q9W4S7|P91664	-	o	-	-	-	v	-
Q9VT33|Q8IMI0	o	-	-	-	-	v	-
Q9VI10|Q9W2P5	o	-	-	-	-	v	-
24657019|Q9W3Y1	o	-	-	-	-	v	-
Q95RA8|Q9VW46	o	-	-	-	-	v	-
O44437|P13060	o	-	-	-	-	v	-
Q9V7F2|17137758	-	-	-	-	-	v	o
Q9VQV0|P54357	o	-	-	-	-	v	-
Q9VZ45|Q564D5	o	-	-	-	-	v	-
24650435|Q9W0R7	-	o	-	-	-	v	-
O77430|Q9VDE3	o	-	-	-	-	v	o
Q9VUN3|Q9VRV8	o	-	-	-	-	v	-
Q9VCY3|Q9XZ58	o	-	-	-	-	v	-
Q9V7F2|Q9VGC7	-	-	-	-	-	v	o
Q9VKW3|P53034	o	-	-	-	-	v	o
Q9VQV0|Q8MKM1	o	-	-	-	-	v	-
Q9VBY2|Q9VLK1	o	-	-	-	-	v	-
Q9VEG5|Q9VPW2	o	-	-	-	-	v	o
Q9VCD5|Q95NR0	o	-	-	-	-	v	-
P45890|O18413	o	-	-	-	-	v	-
Q9V8V3|Q9VT94	-	-	-	-	-	v	o
Q9XZ59|Q9VAJ2	o	-	o	-	-	v	o
Q9W5B3|Q9XZ56	-	-	o	-	-	v	-
Q9VJI7|Q9VRT7	o	-	-	-	-	v	-
Q8T0U6|Q9VNF9	o	-	o	-	-	v	-
Q9VJQ5|Q9W256	o	-	-	-	-	v	-
O18640|Q8T0S6	-	-	-	-	-	v	o
Q9W158|P16914	o	-	-	-	-	v	-
P09085|P17210	-	-	-	-	-	v	o
P00528|O18683	-	o	-	-	-	v	-
O44226|Q9V8V2	-	-	-	-	-	v	o
Q8ING4|Q9VXE5	-	-	-	-	-	v	o
O76521|24584736	o	-	-	-	-	v	-
Q9I7T6|Q8IR26	o	-	-	-	-	v	-
P09089|O96660	-	-	-	-	-	v	o
Q9V782|O97111	-	-	-	-	-	v	o
Q9VSD3|Q9VC05	o	-	-	-	-	v	-
Q9W325|Q9U622	o	-	-	-	-	v	-
Q9VE52|Q7PLX1	o	-	-	-	-	v	-
Q9V9W0|Q9VB23	-	o	-	-	-	v	-
P49071|O61443	o	-	-	-	-	v	o
Q6YN46|Q9VPX7	o	-	-	-	-	v	-
O96660|P31009	-	-	-	-	-	v	o
24584926|Q9W352	-	-	-	-	-	v	o
P23696|Q9W414	o	-	-	-	-	v	-
Q94518|Q9VY35	-	-	-	-	-	v	o
Q9V830|Q9BIR7	o	-	-	-	-	v	-
Q9VR90|Q541G5	o	-	-	-	-	v	-
Q9VSD3|Q9V5D4	o	-	-	-	-	v	-
Q8INQ9|Q9V428	-	-	-	-	-	v	o
Q7KR74|Q9XZ61	-	-	-	-	-	v	o
O18353|Q9VTW5	-	-	o	-	-	v	-

Q9VR35|Q9VSB6	-	-	-	-	-	v	o
Q9VX98|Q9W445	o	-	-	-	-	v	-
Q9V5C6|P12881	o	-	-	-	-	v	-
Q27350|Q05201	-	-	-	-	-	v	o
Q9V830|Q5BI92	o	-	-	-	-	v	-
Q9VHA8|Q9VG58	o	-	-	-	o	v	-
Q24547|Q541G5	o	-	-	-	-	v	-
24583408|Q9W2P5	o	-	-	-	-	v	-
Q7KR54|Q94518	o	-	-	-	-	v	o
Q9VE73|Q9VCE7	-	-	-	-	-	v	o
Q8T3Y0|Q9V9A6	-	-	-	-	-	v	o
Q9V3W9|Q9V9J5	o	-	-	-	-	v	-
Q9VHV0|Q9VTZ1	o	-	-	-	-	v	-
Q9VTW6|Q9W2K2	o	-	-	-	-	v	-
Q9V3J4|Q8MKS2	o	-	-	-	-	v	-
P00528|Q08012	-	o	-	-	-	v	-
P46824|P17210	-	-	-	-	-	v	o
P48456|P49258	o	-	o	-	-	v	-
P48456|P48451	o	-	o	-	-	v	-
P39736|Q9W0R0	o	-	-	-	-	v	-
Q9NJH0|O96827	o	-	-	-	-	v	-
Q8T0S6|Q9VZF4	-	-	-	-	-	v	o
P07909|Q9VHC7	-	-	-	-	-	v	o
Q9VIP1|P49906	o	-	o	-	-	v	-
O18353|P29673	-	-	o	-	-	v	-
P17886|P39736	o	-	-	-	-	v	-
Q9VB62|P26019	o	-	-	-	-	v	-
Q9V859|Q9VZS3	o	-	-	-	-	v	-
Q9XTL2|Q960X8	o	-	-	-	-	v	-
Q9VH07|Q9V3K3	o	-	-	-	-	v	-
Q9VSI1|Q9VP77	o	-	-	-	-	v	-
Q9VQV0|P49258	o	-	-	-	-	v	-
P49258|P54357	o	-	-	-	-	v	-
O77430|Q9VZF4	o	-	-	-	-	v	o
Q9W1Q8|O76863	o	-	-	-	-	v	-
Q86B73|Q9VRP1	o	-	-	-	-	v	-
P02828|Q9V3E9	o	-	-	-	-	v	o
Q9VLK1|Q9V832	o	-	-	-	-	v	-
Q7PL59|Q9VE37	o	-	-	-	-	v	-
Q9XZ68|Q9VN77	o	-	-	-	-	v	-
Q9V5D4|Q9W4N8	o	-	-	-	-	v	-
Q9VKW3|Q9VX15	o	-	-	-	-	v	o
Q9Y0Y5|Q9W0B8	-	-	-	-	-	v	o
Q540V5|Q9VXD0	o	-	-	-	-	v	-
Q9VR29|P13060	o	-	-	-	-	v	-
Q9VAJ2|Q9VJI6	-	-	-	-	-	v	o
Q9VCD5|Q9V478	o	-	-	-	-	v	-
Q94535|Q24562	-	-	o	-	-	v	o
Q53YH7|Q541C2	-	-	-	-	o	v	-
24638774|P49258	o	-	-	-	-	v	-
Q8IRV8|Q9VZK8	-	-	-	-	-	v	o
Q9VTF9|24650097	o	-	-	-	-	v	o
Q9W3N6|Q9VTX7	-	-	-	-	-	v	o
P54359|Q9VXD0	o	-	-	-	-	v	-
Q8IQN4|Q8ING4	-	-	-	-	-	v	o
Q9VJI7|Q9W2P5	o	-	-	-	-	v	-
Q7KVL7|Q9VPX5	o	-	-	-	-	v	-
Q9V830|Q9VG58	o	-	-	-	-	v	-
Q9VXE8|Q9VL00	-	-	-	-	-	v	o
P53501|Q9I7J0	o	-	-	-	-	v	-

P19018|Q02427	-	-	-	-	-	v	o
Q9VN89|Q7KR73	-	-	-	-	-	v	o
Q4PIY5|Q9VUF3	o	-	-	-	-	v	-
Q9VFV9|P29845	o	-	-	-	o	v	-
Q9VI10|24583408	o	-	-	-	-	v	-
Q8IMS8|P16914	o	-	-	-	-	v	o
Q8MKM1|P54357	o	-	-	-	-	v	-
P32392|P25843	o	-	-	-	-	v	-
Q8MKM1|P49258	o	-	-	-	-	v	-
Q9VTW3|O18353	-	-	o	-	-	v	-
Q540X3|Q9VPX7	o	-	-	-	-	v	-
Q9VBI1|Q9W0I7	o	-	-	-	-	v	-
Q7KR74|Q9V3P6	-	-	-	-	-	v	o
Q9VJ37|O18353	-	-	o	-	-	v	-
Q9VM68|Q02926	-	-	-	-	-	v	o
P52654|Q9W5B9	o	-	-	-	-	v	-
Q9VKI8|Q5BI20	o	-	-	-	-	v	-
Q9VGU7|Q9VZF2	-	-	-	-	-	v	o
P02828|Q9V9C5	o	-	-	-	-	v	-
Q9W256|P49258	o	-	-	-	-	v	-
Q9Y0X3|Q24087	o	-	-	-	-	v	-
Q9VPR2|Q94518	-	-	-	-	-	v	o
O76521|Q9VST0	-	-	-	-	-	v	o
Q9VSY2|Q9VSH4	-	-	-	-	-	v	o
P48603|Q9W2N0	o	-	-	-	-	v	-
Q9V3X6|Q9VDW6	-	-	-	-	-	v	o
P23696|Q9W5W6	o	-	-	-	-	v	-
Q9V7H8|O61443	o	-	-	-	-	v	-
P00528|Q9W255	-	o	-	-	-	v	-
P53034|Q9VX15	o	-	-	-	-	v	-
Q9XZ68|Q9W501	o	-	-	-	-	v	-
Q9VMS5|Q9VAJ2	o	-	o	-	-	v	o
Q9V4M7|Q9VJC7	-	-	-	-	o	v	-
P18824|Q9W0P4	-	-	-	-	-	v	o
Q9I7L0|Q7KSH7	-	-	-	-	-	v	o
P02828|Q9VPN5	o	-	-	-	-	v	o
Q9VUF3|Q5U0Y2	o	-	-	-	-	v	-
Q9VTU4|Q9W2D9	-	-	-	-	-	v	o
Q9VV77|Q9W523	-	o	-	-	-	v	-
Q8T6B9|Q540W2	-	-	-	-	-	v	o
Q9VCZ8|Q9VW56	o	-	-	-	-	v	-
Q9W2E3|Q9VJQ5	o	-	-	-	-	v	-
Q9VIZ0|Q9VMX3	o	-	-	-	-	v	-
Q8IME3|Q8INP0	-	-	-	-	-	v	o
Q9VI10|Q9VRT7	o	-	-	-	-	v	-
Q24297|Q9VRT7	o	-	-	-	-	v	-
Q9VWR1|P83967	o	-	-	-	-	v	-
P08182|P08181	o	-	o	-	-	v	-
Q8IR26|P52486	o	-	-	-	-	v	-
O46085|24644592	o	-	-	-	-	v	-
Q9VQQ6|Q9VLQ9	-	-	-	-	-	v	o
Q9VZQ0|Q9VTQ9	o	-	-	-	-	v	-
Q9W3N6|O44424	-	-	-	-	-	v	o
P40427|O46339	-	-	-	-	-	v	o
P53501|P48601	o	-	-	-	-	v	-
Q05825|24647490	o	-	-	-	-	v	-
Q24087|P28518	o	-	-	-	-	v	-
Q9VR90|Q9VHB5	o	-	-	-	-	v	-
Q9VJD4|Q9W227	o	-	-	-	-	v	-
Q9W074|Q9VQQ6	o	-	-	-	-	v	-

	P23757|Q9W3Z2	-	-	-	-	-	v	o
	Q9VW57|Q9W579	-	-	-	-	-	v	o
	Q9VRT7|Q9W2P5	o	-	-	-	-	v	-
	O46085|24582219	o	-	-	-	-	v	-
	P39769|Q9VV77	-	o	-	-	-	v	-
	Q9VST4|Q9W0K4	-	-	-	-	-	v	o
	O46085|Q9VPB8	o	-	-	-	-	v	-
	P41374|24647050	o	-	-	-	-	v	-
	Q9V7W9|Q4PIY5	-	-	o	-	-	v	-
	Q9VXM1|P48609	o	o	o	-	-	v	-
	P17886|Q9W0R0	o	-	-	-	-	v	-
	Q9I7T6|Q9VXP1	o	-	-	-	-	v	-
	Q9VJI7|Q24297	o	-	-	-	-	v	-
	Q9VEQ1|Q86R94	-	-	-	-	-	v	o
	Q9VAP7|Q7KVX1	o	-	o	-	-	v	o
	Q04787|Q9VSK2	-	-	-	-	-	v	o
	Q9V765|Q9VTC4	o	-	-	-	-	v	-
	Q9VJI7|24583408	o	-	-	-	-	v	-
	O18640|P40301	o	-	-	-	-	v	-
	Q24216|P11147	-	o	-	-	-	v	-
	Q9V6U8|Q9VTE9	o	-	-	-	-	v	-
	O46036|Q9VUL0	o	-	-	-	-	v	-
	O77051|Q24318	-	o	o	-	-	v	-
	Q9V469|Q9V3Z4	o	-	-	-	-	v	-
	Q9V9A3|24650924	o	-	-	-	-	v	o
	P15330|Q03017	-	o	o	-	-	v	-
	Q9VI10|Q24297	o	-	-	-	-	v	o
	Q8IQN4|Q9W4Z9	o	-	-	-	-	v	-
	Q9VSU2|17136536	-	-	o	-	-	v	-
	Q9VPV7|Q9VPR2	-	-	-	-	-	v	o
	Q9VXM1|Q9V7R5	o	o	o	-	-	v	-
	Q9VJD4|Q9VHB6	-	-	-	-	-	v	o
	Q8SXG7|Q9VAT3	o	-	-	-	-	v	-
	Q9VR35|Q9W5B3	-	-	-	-	-	v	o
	O18335|Q9V8V3	-	-	-	-	-	v	o
	Q9VHG0|Q9VRV6	-	-	-	-	-	v	o
	Q9VW53|Q9V7H9	o	-	-	-	-	v	-
C.elegans	Q09289|Q09444	-	-	-	-	-	o	v
	O62332|Q20084	o	-	-	-	-	-	v
	O16313|Q9XUE7	-	-	-	-	-	o	v
	Q17878|P30642	-	-	-	-	-	o	v
	P46941|Q27475	o	-	-	-	-	-	v
	O17666|O16305	-	-	-	-	-	o	v
	32566959|Q10454	-	-	-	-	-	o	v
	Q93647|Q93794	o	-	-	-	-	o	v
	P34605|Q20100	o	-	-	-	-	-	v
	P49029|Q21832	-	-	-	-	-	o	v
	Q09584|Q18409	-	-	-	-	-	o	v
	Q23158|Q17718	-	-	-	-	-	o	v
	O44408|Q20347	o	o	-	-	-	-	v
	Q23158|O16299	-	-	-	-	-	o	v
	P35129|Q9XVR6	-	-	-	-	-	o	v
	Q20130|Q21370	-	-	-	-	-	o	v
	Q9NA30|Q09490	o	-	-	-	-	o	v
	O44451|O45924	o	-	o	-	-	o	v
	Q9GRY9|P91277	-	-	-	-	-	o	v
	Q94166|Q564S5	-	-	-	-	-	o	v
	O45551|Q9XW13	-	-	-	-	-	o	v
	Q9N5D6|Q19503	-	-	-	-	-	o	v
	Q22799|Q965U2	-	-	-	-	-	o	v

Q17878|Q23158	-	-	-	-	-	o	v
P50464|O45865	-	-	-	-	-	o	v
O62068|Q20084	-	-	-	-	-	o	v
Q09542|O45577	o	-	-	-	-	-	v
Q22179|Q22352	-	-	-	-	-	o	v
P90978|Q9U2U0	-	-	o	-	-	o	v
P34540|Q21592	-	-	-	-	-	o	v
Q93716|Q18937	-	-	-	-	-	o	v
P10983|Q17446	o	-	-	-	-	-	v
Q23536|Q9NA30	o	-	-	-	-	o	v
P46577|Q9XW68	o	-	-	-	-	o	v
Q19969|Q9N5V3	o	-	-	-	-	-	v
P34475|P52275	o	-	-	-	-	-	v
Q21734|P39745	o	o	-	-	-	-	v
Q95X41|Q95QA3	-	-	-	-	-	o	v
O17915|Q21021	o	-	-	-	-	-	v
Q9U1T6|Q9XXI0	-	-	-	-	-	o	v
O44175|P34429	o	-	-	-	-	o	v
Q17385|O45189	-	-	-	-	-	o	v
O45503|Q21746	-	-	-	-	-	o	v
Q17385|Q20084	-	-	-	-	-	o	v
P46941|Q23671	o	-	-	-	-	-	v
Q95QZ7|Q19584	o	-	-	-	-	o	v
Q9XVS2|Q20414	-	-	-	-	-	o	v
Q19325|Q19555	o	-	-	-	-	-	v
Q18846|Q17446	o	o	-	-	-	-	v
Q21215|Q95QA3	-	-	-	-	-	o	v
P90980|Q9NAN2	-	-	-	-	-	o	v
Q8I7I0|Q9XXI0	-	-	-	-	-	o	v
Q9UAQ6|O17915	o	-	-	-	-	-	v
P02567|P34475	o	-	-	-	-	-	v
Q9XWH0|Q21776	o	-	o	-	-	o	v
O17915|O01583	o	-	-	-	-	-	v
O62195|17554366	-	-	-	-	-	o	v
Q23158|Q23049	-	-	-	-	-	o	v
P34574|P90978	-	-	-	-	-	o	v
Q9N4H7|O62246	-	-	-	-	-	o	v
O18688|O17915	-	-	-	-	-	o	v
O17915|O17244	o	-	-	-	-	-	v
Q22631|Q20577	-	-	-	-	-	o	v
O17894|Q564S5	-	-	-	-	-	o	v
Q9XVF7|Q95YF3	o	-	-	o	-	-	v
Q9XXU4|O44820	-	-	-	-	-	o	v
Q10577|Q19335	o	-	-	-	-	-	v
Q7JK76|P14792	o	-	-	-	-	-	v
P34568|Q17850	-	-	-	-	-	o	v
P91563|Q23158	-	-	-	-	-	o	v
Q9N537|P48727	-	-	o	-	-	o	v
Q09506|O44326	-	-	-	-	-	o	v
O61955|Q9XW13	-	-	-	-	-	o	v
Q9XU10|Q9TVL8	o	-	o	-	-	-	v
Q19266|Q9NAN2	-	-	-	-	-	o	v
Q21693|Q9XW13	-	-	-	-	-	o	v
O76630|Q93568	-	-	-	-	-	o	v
Q10577|Q22469	o	-	-	-	-	o	v
Q9U3S0|P41886	-	-	-	-	-	o	v
P45897|P51403	-	-	-	-	-	o	v
Q23356|Q21215	-	-	-	-	-	o	v
P30625|P21137	o	-	-	-	-	o	v
Q9N2K7|P91276	-	-	-	-	-	o	v

Q18786|P34659	o	-	-	-	-	o	v
P10983|Q9U9Y8	o	-	-	-	-	-	v
Q966C6|Q17359	-	-	-	-	-	o	v
Q17446|Q9TZ16	o	-	-	-	-	o	v
Q22631|Q9XXI0	-	-	-	-	-	o	v
P53016|Q18547	o	-	-	-	-	o	v
Q9XU77|Q20084	-	-	-	-	-	o	v
Q21993|Q9N5M2	o	-	-	-	-	-	v
Q20347|Q8WQG9	o	o	-	-	-	-	v
Q21431|Q17446	o	-	-	-	-	-	v
Q8I7I0|Q20577	-	-	-	-	-	o	v
Q09289|25151723	-	-	-	-	-	o	v
Q9NES0|Q9GYJ9	-	-	-	-	-	o	v
P45896|17554366	-	-	-	-	-	o	v
Q23158|Q9TZA6	-	-	-	-	-	o	v
O18158|Q17446	o	-	-	-	-	-	v
P46941|O45539	-	o	-	-	-	-	v
O01504|P91913	o	-	-	-	-	-	v
P46577|O01426	o	-	-	-	-	o	v
Q9XXA2|Q965X6	-	-	-	-	-	o	v
Q19969|Q21443	-	-	-	-	-	o	v
Q9U2D9|Q17446	o	-	-	-	-	-	v
Q9XU77|Q17359	-	-	-	-	-	o	v
Q22469|Q19335	o	-	-	-	-	-	v
Q9N3Y1|Q18776	-	-	-	-	-	o	v
P52275|Q9XUS3	-	-	-	-	-	o	v
Q9XU77|P34420	-	-	-	-	-	o	v
O01894|Q19584	o	-	-	-	-	o	v
Q93156|Q9XWH0	-	-	-	-	-	o	v
P34540|14018161	-	-	-	-	-	o	v
Q09497|P50464	-	-	-	-	-	o	v
Q9XWU4|Q9BKS1	-	-	-	-	-	o	v
P51875|Q19955	-	-	o	-	-	o	v
Q95QW0|Q9XUP3	-	-	-	-	-	o	v
Q22235|Q21746	o	-	-	-	-	o	v
Q09997|17554366	-	-	-	-	-	o	v
Q19019|17554366	-	-	-	-	-	o	v
Q9XTG7|Q95XS8	o	-	-	-	-	-	v
Q86S66|Q18885	o	-	-	-	-	o	v

Note: "v" represents that conserved PPIs are from the corresponding species, "o" represents that homologous PPIs exist in the corresponding species, "-" represents that no homologous PPIs are found in the corresponding species.
