# Supplementary material for: Comparison of protein interaction networks reveals species conservation and divergence
Source: BMC Bioinformatics. 2006 Oct 17;7:457. doi: 10.1186/1471-2105-7-457 (PMC1630707; doi:10.1186/1471-2105-7-457)
Supplement: Additional file 2 — Predicted PPIs. The list of predicted PPIs derived from the analysis. [file 1471-2105-7-457-S2.pdf]

	PPI	Refence species	Refence PPI
S.cerevisiae	P32776|Q00578	*H.sapiens*	Q6I9Y7|Q53QM0
	P06839|Q12004	*H.sapiens*	P18074|Q13889
	P32776|P06839	*H.sapiens*	Q6I9Y7|P18074
	Q04673|Q00578	*H.sapiens*	Q13888|Q53QM0
	P40089|P54999	*D.melanogaster*	Q9VRT7|Q24297
	P06839|P07276	*H.sapiens*	P28715|P18074
	Q54AF5|P09457	*E.coli*	P00837|P00831
	Q04673|Q12004	*H.sapiens*	Q13888|Q13889
	Q04673|P07276	*H.sapiens*	Q13888|P28715
	P53905|P54999	*D.melanogaster*	Q9VJI7|Q24297
	P15873|P32641	*H.sapiens*	P12004|O75943
	P53905|Q12330	*D.melanogaster*	Q9VJI7|Q9VLV5
C.elegans	Q22469|Q19335	*S.cerevisiae*	Q07350|P19736
H.pylori	P55993|P56001	*E.coli*	P00579|P0A7Z4
	P56001|O25676	*E.coli*	P31804|P0A7Z4
M.musculus	Q13873|P37172	*H.sapiens*	P37173|P37023
	423499|Q5NCK7	*S.cerevisiae*	Q00772|Q01389
	P36898|P12644	*H.sapiens*	Q5T7S2|P10600
	Q13873|P43026	*H.sapiens*	P01137|P37173
	P27037|P22004	*H.sapiens*	P37173|P10600
	Q64261|Q4FK45	*H.sapiens*	P24941|P20248
	P37172|P43026	*H.sapiens*	P01137|P37023
	P63139|Q62725	*S.cerevisiae*	P13434|Q02516
	Q53Z43|P43026	*H.sapiens*	P01137|P37023
	P27040|P18075	*H.sapiens*	P37173|P10600
	Q53Z43|P27040	*H.sapiens*	P37023|P37173
	P27037|P43026	*H.sapiens*	P01137|P37173
	Q13873|P27040	*H.sapiens*	Q5T7S2|P37173
	P62821|P35279	*S.cerevisiae*	P36018|P36017
	P62821|P20339	*S.cerevisiae*	P36018|P36017
	P27040|P43026	*H.sapiens*	P01137|P37173
	Q13873|P22004	*H.sapiens*	P37173|P10600
	P27037|P27040	*H.sapiens*	Q5T7S2|P37173
	P35279|P61019	*S.cerevisiae*	P36018|P36017
	P12644|P37172	*H.sapiens*	P01137|P37023
	Q53Z43|P18075	*H.sapiens*	P10600|P37023
	P37172|P27040	*H.sapiens*	P37023|P37173
	P30285|Q4FK45	*H.sapiens*	P24941|P20248
	P27037|P37172	*H.sapiens*	P37173|P37023
	P61019|P20339	*S.cerevisiae*	P01123|P07560
	Q13873|Q53Z43	*H.sapiens*	P37173|P37023
	P35279|P20339	*S.cerevisiae*	P36018|P36017
	P37172|P18075	*H.sapiens*	P10600|P37023
	P27037|P18075	*H.sapiens*	P10600|P37173
	P62821|P61019	*S.cerevisiae*	P36018|P36017
	P36898|P27040	*H.sapiens*	Q5T7S2|P37173
	P97377|Q4FK45	*H.sapiens*	P24941|P20248
	P12644|P27040	*H.sapiens*	P10600|P37173
	P37237|P16092	*H.sapiens*	P09038|P11362
	P27037|Q53Z43	*H.sapiens*	P37173|P37023
	P27037|P12644	*H.sapiens*	P37173|P10600
E.coli	P00831|P00855	*S.cerevisiae*	P09457|Q96101
	P00822|P00855	*S.cerevisiae*	P07251|Q96101
D.melanogaster	Q9VI10|24583408	*S.cerevisiae*	P40070|Q06217
	24583408|Q9W2P5	*S.cerevisiae*	P40070|Q06406
	Q9VRT7|24583408	*S.cerevisiae*	P40070|P40089

	Q9VJI7|Q9VI10	*S.cerevisiae*	P53905|Q06217
	P39736|Q9W0R0	*S.cerevisiae*	Q03654|P28004
	Q9VJI7|Q9W2P5	*S.cerevisiae*	P53905|Q06406
	Q9VQV0|P49258	*S.cerevisiae*	P06787|P53141
	Q9VI10|Q9W2P5	*S.cerevisiae*	Q06406|Q06217
	P53034|Q9VX15	*S.cerevisiae*	P38630|P38251
	P49258|P54357	*S.cerevisiae*	P06787|P53141
	Q9VQV0|P54357	*S.cerevisiae*	P06787|P53141
	Q540V5|Q9VXD0	*S.cerevisiae*	P32468|P32457
	Q24087|P28518	*S.cerevisiae*	P06777|P28519
	Q9VLV5|Q9VI10	*S.cerevisiae*	Q06217|Q12330
H.sapiens	Q6FG41|P18848	*M.musculus*	P01101|Q61328
	P62258|P31946	*S.cerevisiae*	P29311|P34730
	Q04917|P31946	*S.cerevisiae*	P29311|P34730
	P24941|Q6FI05	*M.musculus*	Q64261|Q564P6
	Q6FH52|P62258	*S.cerevisiae*	P29311|P34730
	P19784|Q4VX47	*S.cerevisiae*	P15790|P43639
	Q6ICQ9|P05412	*M.musculus*	P01101|Q569U6
	P06493|P38936	*M.musculus*	Q564P6|P97377
	P06493|P46527	*M.musculus*	Q564P6|P30285
	Q6I9Y7|Q13889	*S.cerevisiae*	P32776|Q12004
	Q567U5|P31946	*S.cerevisiae*	P29311|P34730
	P19784|P68400	*S.cerevisiae*	P15790|P19454
	Q04917|Q6FH52	*S.cerevisiae*	P29311|P34730
	Q6FI00|Q00534	*M.musculus*	Q790L7|P97377
	Q567U5|P62258	*S.cerevisiae*	P29311|P34730
	Q6FI00|P11802	*M.musculus*	Q790L7|P30285
	P63208|P62877	*S.cerevisiae*	P52286|Q08273
	Q6FI00|P46527	*M.musculus*	Q790L7|Q564P6
	P38936|P20248	*M.musculus*	Q790L7|Q564P6
	P20248|P11802	*M.musculus*	Q790L7|P30285
	Q04917|P62258	*S.cerevisiae*	P29311|P34730
	Q5U035|P38936	*M.musculus*	Q564P6|Q790L7
	Q6FI00|P38936	*M.musculus*	Q790L7|Q564P6
	Q567U5|Q6FH52	*S.cerevisiae*	P29311|P34730
	Q6FI05|P06493	*M.musculus*	Q564P6|P30285
	Q13156|P43351	*S.cerevisiae*	P06778|P26754
	P18848|Q6ICQ9	*M.musculus*	Q61328|P01101
	P13862|Q4VX47	*S.cerevisiae*	P38930|P43639
	P17080|P62826	*S.cerevisiae*	P32835|P32836
	Q04917|Q567U5	*S.cerevisiae*	P29311|P34730
	P24941|Q5U035	*M.musculus*	Q64261|Q790L7
	Q5U035|Q00534	*M.musculus*	P97377|Q790L7
	Q6FH52|P31946	*S.cerevisiae*	P29311|P34730
	P20248|Q00534	*M.musculus*	Q790L7|P97377
