# Supplementary material for: Comparison of protein interaction networks reveals species conservation and divergence
Source: BMC Bioinformatics. 2006 Oct 17;7:457. doi: 10.1186/1471-2105-7-457 (PMC1630707; doi:10.1186/1471-2105-7-457)
Supplement: Additional file 3 — Function prediction. The list of predicted function annotations derived from the analysis. [file 1471-2105-7-457-S3.pdf]

Protein	GO annotation	Protein	GO annotation
O95750	0009887	Q6I9Y7	0005675
O95750	0008283	Q6I9Y7	0006950
O95750	0007267	Q6I9Y7	0006289
P22455	0009887	Q6I9Y7	0005667
P22455	0008283	Q13888	0006974
P22455	0007267	Q13888	0005654
P11487	0009887	Q13888	0016591
P10767	0009887	Q13888	0006281
P05230	0009887	Q13888	0005675
P31371	0009887	Q13888	0006950
P22607	0009887	Q13888	0006289
P22607	0008283	Q13888	0005667
P22607	0007267	Q13889	0006974
P21802	0009887	Q13889	0005654
P21802	0008283	Q13889	0016591
P21802	0007267	Q13889	0006281
P08620	0009887	Q13889	0005675
Q6FGV5	0009887	Q13889	0006950
Q6FGV5	0008283	Q13889	0006289
Q6FGV5	0007267	Q13889	0005667
P12034	0009887	P24870	0051325
P11362	0009887	P24870	0000278
P11362	0008283	P24870	0051329
P11362	0007267	P00546	0051325
P21802	0009887	P00546	0000074
P21802	0008283	P00546	0051244
P21802	0007267	P00546	0000278
P16092	0008283	P00546	0051329
Q61851	0009887	P24868	0051325
Q61851	0008283	P24868	0000278
Q61851	0007267	P24868	0051329
Q00534	0000278	P24871	0051325
P14635	0000278	P24871	0000278
P11802	0000074	P24871	0051329
P11802	0051244	P24869	0051325
P11802	0007049	P24869	0000278
P11802	0000278	P24869	0051329
Q14010	0000074	P30283	0051325
Q14010	0051244	P30283	0000278
Q14010	0007049	P30283	0051329
Q14010	0000278	P14635	0051325
Q6FI00	0000278	P14635	0000278
Q6FI05	0007049	P14635	0051329
Q6FI05	0000278	P11802	0051325
P06493	0000278	P11802	0000074
P20248	0000278	P11802	0051244
P24864	0000074	P11802	0007049
P24864	0051244	P11802	0000278
P24864	0007049	P11802	0051329
P24864	0000278	Q6FI00	0051325
Q96TE0	0000074	Q6FI00	0000278
Q96TE0	0051244	Q6FI00	0051329
Q96TE0	0007049	P06493	0051325
Q96TE0	0000278	P06493	0000278
P24941	0000278	P06493	0051329
Q5U035	0000278	P20248	0051325
Q564P6	0007049	P20248	0000278

Q564P6	0000278
Q4FJM2	0000074
Q4FJM2	0051244
Q4FJM2	0007049
Q4FJM2	0000278
Q64261	0000074
Q64261	0051244
Q64261	0000278
P30285	0000278
P97377	0000074
P97377	0051244
P97377	0000278
Q790L7	0000278
P43063	0000074
P43063	0051244
P43063	0000278
Q4FK45	0007049
Q4FK45	0000278
882391*	0007167
882391*	0007178
Q4VAV9	0007167
Q4VAV9	0007178
P12643	0007167
P12643	0007178
P10600	0007167
P10600	0007178
P12644	0007167
P12644	0007178
P22004	0007167
P22004	0007178
P08476	0007167
P08476	0007178
Q13873	0007167
Q13873	0007178
P18075	0007167
P18075	0007178
P03989	0001772
Q30201	0001772
Q5HYM5	0001772
Q5HYM5	0030333
Q53Z42	0001772
Q53Z42	0030333
Q542Z3	0006952
Q542Z3	0009607
Q542Z3	0001772
Q542Z3	0030333
Q542Z3	0006955
Q542Z3	0019882
P04229	0001772
Q6DU50	0001772
Q6DU50	0030333
Q5TK76	0001772
Q5TK76	0030333
Q6IAZ1	0001772
Q6IAZ1	0030333
Q6IAZ1	0019882
Q5SS57	0006952
Q5SS57	0009607
P20248	0051329
P24864	0051325
P24864	0000074
P24864	0051244
P24864	0007049
P24864	0000278
P24864	0051329
P24941	0051325
P24941	0000278
P24941	0051329
Q5U035	0051325
Q5U035	0000278
Q5U035	0051329
P15873	0005663
P15873	0030894
P15873	0006263
P15873	0005694
P32641	0030894
P32641	0005657
P32641	0006263
P32641	0005694
P38630	0030894
P38630	0005657
P38630	0006263
P38630	0005694
P40348	0030894
P40348	0005657
P40348	0006263
P40348	0005694
P40339	0030894
P40339	0005657
P40339	0006263
P40339	0005694
P38629	0030894
P38629	0005657
P38629	0006263
P38629	0005694
P38251	0030894
P38251	0005657
P38251	0006263
P38251	0005694
Q6FHX7	0005663
Q6FHX7	0030894
Q6FHX7	0005657
Q6FHX7	0006263
Q6FHX7	0005694
O75943	0006260
O75943	0005663
O75943	0030894
O75943	0005657
O75943	0006263
O75943	0006259
O75943	0005694
P12004	0005663
P12004	0030894
P12004	0005657
P12004	0006263
P12004	0005694

Q5SS57	0001772
Q5SS57	0030333
Q5SS57	0006955
Q5SS57	0019882
P61769	0001772
Q5Y7D1	0001772
Q546I9	0001772
Q546I9	0030333
Q792Z7	0001772
Q792Z7	0030333
Q7JJ15	0001772
Q7JJ15	0030333
P23150	0001772
P06343	0001772
P01900	0001772
P11609	0001772
P11609	0030333
P01887	0001772
Q598Q1	0007399
Q5VVP4	0007399
P01138	0007399
Q01973	0007399
Q91407	0007399
Q6FH50	0007399
P20181	0007399
Q541P3	0007399
P06839	0005654
P06839	0016591
Q04673	0005654
Q04673	0016591
P07276	0005654
P07276	0016591
P07276	0005675
P07276	0005667
Q12004	0005654
Q12004	0016591
Q12004	0006289
P32776	0005654
P32776	0016591
Q00578	0005654
Q00578	0016591
P28715	0005654
P28715	0016591
P28715	0005675
P28715	0005667
Q53QM0	0005654
Q53QM0	0016591
Q53QM0	0005675
Q53QM0	0005667
P18074	0005654
P18074	0016591
Q6I9Y7	0006974
Q6I9Y7	0005654
Q6I9Y7	0016591
Q6I9Y7	0006281
P26754	0005694
P22336	0005694
P38111	0005694
P78527	0005694
P43351	0005694
P27694	0005694
Q6FHX9	0006310
Q6FHX9	0005694
Q13156	0006950
Q13156	0006974
Q13156	0006281
Q13156	0006310
Q13156	0009719
Q13156	0005694
P38930	0004674
P38930	0016301
P38930	0016773
P38930	0004672
P43639	0004674
P43639	0016301
P43639	0016773
P43639	0004672
Q4VX47	0004674
Q4VX47	0016773
Q4VX47	0004672
P23287	0004722
P14747	0004722
P06787	0008287
P06787	0004722
P06787	0005955
Q53SL0	0008287
Q53SL0	0004722
Q53SL0	0005955
1083499*	0006468
1083499*	0016773
1083499*	0006793
1083499*	0006796
1083499*	0004672
1083499*	0016310
1083499*	0004674
1083499*	0016772
1083499*	0016301
423499*	0006468
423499*	0016773
423499*	0006793
423499*	0006796
423499*	0004672
423499*	0016310
423499*	0004674
423499*	0016772
423499*	0016301
P00546	0000074
Q64261	0000074
P97377	0000074

* GenBank GI numbers
