# Supplementary material for: Comparison of protein interaction networks reveals species conservation and divergence
Source: BMC Bioinformatics. 2006 Oct 17;7:457. doi: 10.1186/1471-2105-7-457 (PMC1630707; doi:10.1186/1471-2105-7-457)
Supplement: Additional file 4 — Ortholog prediction. The list of predicted orthologs derived from the analysis. [file 1471-2105-7-457-S4.pdf]

Ortholog A	Species A	Ortholog B	Species B
P36018	*S.cerevisiae*	P35279	*M.musculus*
P01123	*S.cerevisiae*	P62821	*M.musculus*
P01123	*S.cerevisiae*	P20339	*M.musculus*
P36019	*S.cerevisiae*	P61019	*M.musculus*
P38146	*S.cerevisiae*	P20339	*M.musculus*
P51996	*S.cerevisiae*	P20339	*M.musculus*
P36017	*S.cerevisiae*	P61019	*M.musculus*
P38555	*S.cerevisiae*	P61019	*M.musculus*
P38555	*S.cerevisiae*	P62821	*M.musculus*
P36019	*S.cerevisiae*	P35279	*M.musculus*
P07560	*S.cerevisiae*	P20339	*M.musculus*
P01123	*S.cerevisiae*	P61019	*M.musculus*
P36017	*S.cerevisiae*	P35279	*M.musculus*
P36018	*S.cerevisiae*	P20339	*M.musculus*
P38555	*S.cerevisiae*	P35279	*M.musculus*
P51996	*S.cerevisiae*	P61019	*M.musculus*
P32939	*S.cerevisiae*	P61019	*M.musculus*
P01123	*S.cerevisiae*	P35279	*M.musculus*
P38146	*S.cerevisiae*	P35279	*M.musculus*
P36019	*S.cerevisiae*	P62821	*M.musculus*
P39958	*S.cerevisiae*	Q541Z9	*M.musculus*
P07560	*S.cerevisiae*	P61019	*M.musculus*
P32939	*S.cerevisiae*	P62821	*M.musculus*
P36019	*S.cerevisiae*	P20339	*M.musculus*
P51996	*S.cerevisiae*	P35279	*M.musculus*
P32939	*S.cerevisiae*	P35279	*M.musculus*
P36018	*S.cerevisiae*	P61019	*M.musculus*
P36017	*S.cerevisiae*	P62821	*M.musculus*
P36018	*S.cerevisiae*	P62821	*M.musculus*
P36017	*S.cerevisiae*	P20339	*M.musculus*
Q99260	*S.cerevisiae*	P35279	*M.musculus*
P51996	*S.cerevisiae*	P62821	*M.musculus*
P07560	*S.cerevisiae*	P62821	*M.musculus*
P38555	*S.cerevisiae*	P20339	*M.musculus*
Q99260	*S.cerevisiae*	P62821	*M.musculus*
P07560	*S.cerevisiae*	P35279	*M.musculus*
P32864	*S.cerevisiae*	Q541Z9	*M.musculus*
P26754	*S.cerevisiae*	Q13156	*H.sapien*
P30283	*S.cerevisiae*	P24864	*H.sapien*
P00546	*S.cerevisiae*	P06493	*H.sapien*
P15790	*S.cerevisiae*	P68400	*H.sapien*
P24871	*S.cerevisiae*	P20248	*H.sapien*
P38111	*S.cerevisiae*	P78527	*H.sapien*
P32776	*S.cerevisiae*	Q6I9Y7	*H.sapien*
P24868	*S.cerevisiae*	P20248	*H.sapien*
P24869	*S.cerevisiae*	Q6FI00	*H.sapien*
P24870	*S.cerevisiae*	P20248	*H.sapien*
P30283	*S.cerevisiae*	P20248	*H.sapien*
P38930	*S.cerevisiae*	Q4VX47	*H.sapien*
P19454	*S.cerevisiae*	P68400	*H.sapien*
P07276	*S.cerevisiae*	P28715	*H.sapien*
P24869	*S.cerevisiae*	P24864	*H.sapien*
P06839	*S.cerevisiae*	P18074	*H.sapien*
P19454	*S.cerevisiae*	P19784	*H.sapien*
P22336	*S.cerevisiae*	P27694	*H.sapien*
P24871	*S.cerevisiae*	Q6FI00	*H.sapien*
P38930	*S.cerevisiae*	P13862	*H.sapien*

P43639	*S.cerevisiae*	Q4VX47	*H.sapien*
P24870	*S.cerevisiae*	P14635	*H.sapien*
P24868	*S.cerevisiae*	P14635	*H.sapien*
P24868	*S.cerevisiae*	Q6FI00	*H.sapien*
P15790	*S.cerevisiae*	P19784	*H.sapien*
P24870	*S.cerevisiae*	Q6FI00	*H.sapien*
P24869	*S.cerevisiae*	P20248	*H.sapien*
P24869	*S.cerevisiae*	P14635	*H.sapien*
P43639	*S.cerevisiae*	P13862	*H.sapien*
P24871	*S.cerevisiae*	P24864	*H.sapien*
Q04673	*S.cerevisiae*	Q13888	*H.sapien*
P24868	*S.cerevisiae*	P24864	*H.sapien*
P25454	*S.cerevisiae*	Q6FHX9	*H.sapien*
P07251	*S.cerevisiae*	P00822	*E.coli*
Q54AF5	*S.cerevisiae*	P00837	*E.coli*
P07251	*S.cerevisiae*	P00824	*E.coli*
P12398	*S.cerevisiae*	P0A6Y8	*E.coli*
P35191	*S.cerevisiae*	P08622	*E.coli*
P00830	*S.cerevisiae*	P00822	*E.coli*
P38523	*S.cerevisiae*	P09372	*E.coli*
P00830	*S.cerevisiae*	P00824	*E.coli*
P09457	*S.cerevisiae*	P00831	*E.coli*
P27466	*S.cerevisiae*	24638774*	*D.melanogaster*
P53141	*S.cerevisiae*	P54357	*D.melanogaster*
P23287	*S.cerevisiae*	P48456	*D.melanogaster*
Q12330	*S.cerevisiae*	Q9VLV5	*D.melanogaster*
P14747	*S.cerevisiae*	P48456	*D.melanogaster*
Q02516	*S.cerevisiae*	Q9W256	*D.melanogaster*
P53141	*S.cerevisiae*	Q9VQV0	*D.melanogaster*
P13434	*S.cerevisiae*	Q9VJQ5	*D.melanogaster*
P13434	*S.cerevisiae*	Q8ST61	*D.melanogaster*
P19524	*S.cerevisiae*	Q8MKM1	*D.melanogaster*
P54999	*S.cerevisiae*	Q24297	*D.melanogaster*
Q06217	*S.cerevisiae*	Q9VI10	*D.melanogaster*
P06787	*S.cerevisiae*	P49258	*D.melanogaster*
P03989	*H.sapien*	Q7JJ15	*M.musculus*
Q5TK76	*H.sapien*	P11609	*M.musculus*
Q30201	*H.sapien*	P11609	*M.musculus*
Q5SS57	*H.sapien*	P11609	*M.musculus*
P37023	*H.sapien*	Q53Z43	*M.musculus*
Q96TE0	*H.sapien*	Q564P6	*M.musculus*
P01137	*H.sapien*	P22004	*M.musculus*
Q542Z3	*H.sapien*	P11609	*M.musculus*
P01137	*H.sapien*	P43026	*M.musculus*
Q6ICQ9	*H.sapien*	P01101	*M.musculus*
Q00534	*H.sapien*	P30285	*M.musculus*
Q546I9	*H.sapien*	P11609	*M.musculus*
P20248	*H.sapien*	Q4FK45	*M.musculus*
Q5TK76	*H.sapien*	Q7JJ15	*M.musculus*
Q4VAV9	*H.sapien*	P22004	*M.musculus*
Q30201	*H.sapien*	Q7JJ15	*M.musculus*
Q00534	*H.sapien*	Q64261	*M.musculus*
Q5SS57	*H.sapien*	Q7JJ15	*M.musculus*
P24941	*H.sapien*	P97377	*M.musculus*
Q4VAV9	*H.sapien*	P43026	*M.musculus*
P10600	*H.sapien*	P22004	*M.musculus*
Q542Z3	*H.sapien*	Q7JJ15	*M.musculus*
Q6FI05	*H.sapien*	Q564P6	*M.musculus*

P06493	*H.sapien*	Q64261	*M.musculus*
Q53Z42	*H.sapien*	P11609	*M.musculus*
Q5U035	*H.sapien*	Q4FK45	*M.musculus*
Q6FI00	*H.sapien*	Q4FK45	*M.musculus*
P10600	*H.sapien*	P43026	*M.musculus*
Q546I9	*H.sapien*	Q7JJ15	*M.musculus*
P24864	*H.sapien*	Q4FK45	*M.musculus*
Q30201	*H.sapien*	P01900	*M.musculus*
Q5TK76	*H.sapien*	P01900	*M.musculus*
Q5T7S2	*H.sapien*	P37172	*M.musculus*
P06493	*H.sapien*	P30285	*M.musculus*
Q5SS57	*H.sapien*	P01900	*M.musculus*
P11802	*H.sapien*	Q64261	*M.musculus*
P05412	*H.sapien*	Q569U6	*M.musculus*
Q30201	*H.sapien*	Q792Z7	*M.musculus*
Q5TK76	*H.sapien*	Q792Z7	*M.musculus*
Q5SS57	*H.sapien*	Q792Z7	*M.musculus*
Q542Z3	*H.sapien*	P01900	*M.musculus*
Q00534	*H.sapien*	P97377	*M.musculus*
Q5HYM5	*H.sapien*	P11609	*M.musculus*
Q542Z3	*H.sapien*	Q792Z7	*M.musculus*
Q546I9	*H.sapien*	P01900	*M.musculus*
P01137	*H.sapien*	P12644	*M.musculus*
P06493	*H.sapien*	P97377	*M.musculus*
Q6DU50	*H.sapien*	P11609	*M.musculus*
P37173	*H.sapien*	Q13873	*M.musculus*
P37023	*H.sapien*	P37172	*M.musculus*
P11802	*H.sapien*	P30285	*M.musculus*
P37023	*H.sapien*	P36898	*M.musculus*
Q53Z42	*H.sapien*	P01900	*M.musculus*
Q4VAV9	*H.sapien*	P12644	*M.musculus*
Q5T7S2	*H.sapien*	P27040	*M.musculus*
Q5HYM5	*H.sapien*	Q7JJ15	*M.musculus*
P37173	*H.sapien*	P27037	*M.musculus*
Q53Z42	*H.sapien*	Q792Z7	*M.musculus*
P10600	*H.sapien*	P12644	*M.musculus*
Q5T7S2	*H.sapien*	P36898	*M.musculus*
Q6DU50	*H.sapien*	Q7JJ15	*M.musculus*
P01137	*H.sapien*	P18075	*M.musculus*
Q5U035	*H.sapien*	Q790L7	*M.musculus*
P03989	*H.sapien*	P11609	*M.musculus*
P24941	*H.sapien*	P30285	*M.musculus*
Q6FG41	*H.sapien*	P13346	*M.musculus*
Q5HYM5	*H.sapien*	P01900	*M.musculus*
Q5T7S2	*H.sapien*	Q53Z43	*M.musculus*
P20248	*H.sapien*	Q790L7	*M.musculus*
Q5HYM5	*H.sapien*	Q792Z7	*M.musculus*
Q4VAV9	*H.sapien*	P18075	*M.musculus*
P37173	*H.sapien*	P27040	*M.musculus*
Q6DU50	*H.sapien*	P01900	*M.musculus*
P24941	*H.sapien*	Q64261	*M.musculus*
P10600	*H.sapien*	P18075	*M.musculus*
P05412	*H.sapien*	Q52L79	*M.musculus*
O25806	*H.pylori*	P0A8V2	*E.coli*
O25806	*H.pylori*	P0A8T7	*E.coli*

* GenBank GI numbers
